# Genome-Wide DNA Methylation Analysis Predicts an Epigenetic Switch for GATA Factor Expression in Endometriosis

**DOI:** 10.1371/journal.pgen.1004158

**Published:** 2014-03-06

**Authors:** Matthew T. Dyson, Damian Roqueiro, Diana Monsivais, C. Mutlu Ercan, Mary Ellen Pavone, David C. Brooks, Toshiyuki Kakinuma, Masanori Ono, Nadereh Jafari, Yang Dai, Serdar E. Bulun

**Affiliations:** 1 Division of Reproductive Biology Research, Dept. Obstetrics and Gynecology, Feinberg School of Medicine, Northwestern University, Chicago, Illinois, United States of America; 2 Laboratory of Computational Functional Genomics, Dept. Bioengineering, University of Illinois at Chicago, Chicago, Illinois, United States of America; Stanford University School of Medicine, United States of America

## Abstract

Endometriosis is a gynecological disease defined by the extrauterine growth of endometrial-like cells that cause chronic pain and infertility. The disease is limited to primates that exhibit spontaneous decidualization, and diseased cells are characterized by significant defects in the steroid-dependent genetic pathways that typify this process. Altered DNA methylation may underlie these defects, but few regions with differential methylation have been implicated in the disease. We mapped genome-wide differences in DNA methylation between healthy human endometrial and endometriotic stromal cells and correlated this with gene expression using an interaction analysis strategy. We identified 42,248 differentially methylated CpGs in endometriosis compared to healthy cells. These extensive differences were not unidirectional, but were focused intragenically and at sites distal to classic CpG islands where methylation status was typically negatively correlated with gene expression. Significant differences in methylation were mapped to 403 genes, which included a disproportionally large number of transcription factors. Furthermore, many of these genes are implicated in the pathology of endometriosis and decidualization. Our results tremendously improve the scope and resolution of differential methylation affecting the HOX gene clusters, nuclear receptor genes, and intriguingly the GATA family of transcription factors. Functional analysis of the GATA family revealed that *GATA2* regulates key genes necessary for the hormone-driven differentiation of healthy stromal cells, but is hypermethylated and repressed in endometriotic cells. *GATA6*, which is hypomethylated and abundant in endometriotic cells, potently blocked hormone sensitivity, repressed *GATA2*, and induced markers of endometriosis when expressed in healthy endometrial cells. The unique epigenetic fingerprint in endometriosis suggests DNA methylation is an integral component of the disease, and identifies a novel role for the GATA family as key regulators of uterine physiology–aberrant DNA methylation in endometriotic cells correlates with a shift in GATA isoform expression that facilitates progesterone resistance and disease progression.

## Author Summary

Women develop endometriosis when endometrial tissue with altered sensitivity to ovarian hormones grows outside the uterus. The persistent survival of these cells results in chronic pelvic pain and infertility. Although the origin of the disease remains a mystery, it only occurs in women and menstruating primates, suggesting that the unique evolution behind primate uterine development and menstruation are linked to the disease. Epigenetic defects affecting the uterine physiological response to ovarian hormones are also involved in endometriosis, and several genes implicated in disease progression are differentially methylated. Here we compared DNA methylation with gene expression in endometriosis using large-scale arrays. By comparing healthy and diseased cells treated with or without hormones to mimic part of the menstrual cycle, we uncovered many differentially methylated genes with defective expression in endometriosis that also regulate the hormone-dependent aspects of menstruation. In addition to expanding our understanding of how methylation affects endometriosis many fold, this also led us to propose an epigenetic switch that permits GATA6 expression in endometriosis instead of GATA2, and this switch promotes the aberrant expression of many of the genes seen in endometriosis. Our work provides novel unifying insight into the cause and development of endometriosis.

## Introduction

Endometriosis is a painful and chronic gynecological disease that affects approximately 10% of reproductive-age women, causing infertility and development of adhesions due to the extrauterine growth of endometrium-like tissue [1], [2]. The molecular cause of endometriosis is multifactorial, with disease occurrence and severity influenced by heritable components as well as environmental and lifestyle factors [3], [4]. The cellular origin of endometriosis has been elusive, and several different models have been proposed to account for the many manifestations of the disease [5]. Of these, Sampson's model of retrograde menstruation remains most widely accepted, because the frequent occurrence of menstrual reflux explains the more common distribution of endometriosis to the ovaries and pelvic peritoneum, and because explanted endometrial tissue can give rise to endometriotic lesions [5]–[7]. Sampson's model was also remarkably intuitive, as we now observe endometriosis to be medically, historically, and evolutionarily linked to menstruation. Yet this model fails explain why only 10% of women develop endometriosis when most experience retrograde menstruation, nor can it explain instances of endometriosis that arise independently of menstruation [8], [9]. Emera and Wagner provided clarity by proposing that menstruation is a mechanistic consequence of the evolution of hormone-induced spontaneous differentiation of the endometrium [10]. We would extend this model to the molecular level by suggesting that changes in the unique genetic regulatory networks controlling this hormonal trigger in menstruating primates permit the development of endometriosis.

In most placental mammals, the differentiation (decidualization) of the endometrial stroma into the decidual cells of pregnancy is induced by the implanting blastocyst [11], [12]; however, primates that menstruate initiate decidualization through an evolutionarily unique mechanism: the post-ovulatory rise in maternal progesterone [10], [13], [14]. Consequently, decidualization is triggered in women with every ovulatory cycle independent of pregnancy. Continued development and maintenance of the decidua is dependent on progesterone, and hormone withdrawal in the absence of a pregnancy provokes targeted apoptosis and eventual shedding of the superficial endometrium [15]. This physiological response is blunted in endometriosis. In contrast to healthy tissue, endometriotic tissues are progesterone-insensitive and resistant to apoptosis [16], [17], and many of the pathways utilized in progesterone-dependent decidualization are dysregulated in endometriotic lesions [18]–[20]. This suggests that alterations in the steroid-governed pathways unique to spontaneous decidualization underlie the pathogenesis of endometriosis. Intriguingly, the molecular characterization of diseased cells suggests that epigenetic defects strongly affect these pathways.

DNA methylation serves as a critical regulator of gene expression, and global differences in DNA methylation affect multiple aspects of development and disease. Endometriotic cells express variable levels of the DNA methyltransferase enzymes (DNMTs), which introduce and maintain DNA methylation on the C5 position of cytosine in CpG dinucleotides [21]. Abnormal DNA methylation in endometriosis affects the expression of several genes, including homeobox A10 (*HOXA10*), estrogen receptor beta (*ESR2*), steroidogenic factor 1 (*NR5A1*), and aromatase (*CYP19A1*), which alter steroid signaling and responsiveness, and are critically involved in development and decidualization [3], [4],[22]–[24]. While we and others have uncovered several individual genes with altered DNA methylation in endometriosis, the global profile of DNA methylation in endometriosis has not been characterized with the granularity necessary to detect many gene-specific methylation differences. We hypothesize that highly focused deviations in stromal cell DNA methylation, either inherited or acquired, affect key genes involved in spontaneous decidualization and contribute to the progression of endometriosis. These defects alter gene expression in stromal cells and their response to steroid hormone signaling during the menstrual cycle. Here, we used Illumina's high resolution HumanMethylation450 beadchips in conjunction with gene expression arrays to compare ovarian endometriotic stromal cells (OSIS) and healthy endometrial stromal cells (EIUM) treated with or without decidualizing stimuli.

## Results

### Gene expression analysis identifies differences based on disease and in hormone responsivity

**Figure 1:**
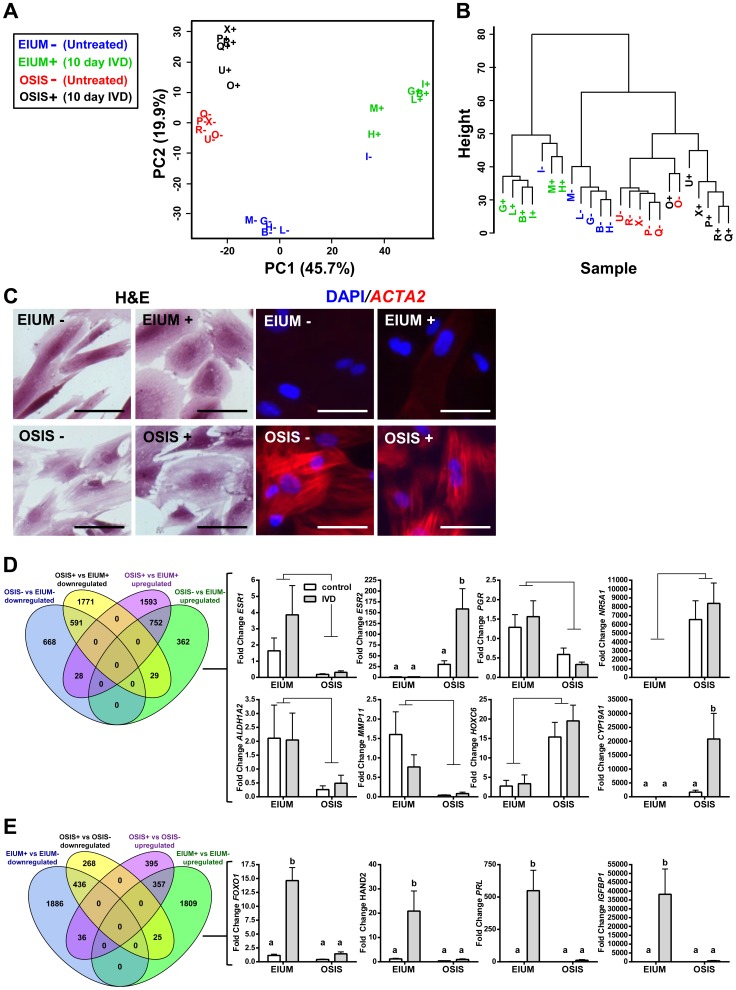
Gene expression differences in healthy and endometriotic stroma. Gene expression in normal endometrial stromal cells (EIUM; n = 6) and endometriotic stromal cells (OSIS; n = 6) treated with (+) or without (−) IVD was examined by (A) principal component analysis and (B) hierarchical clustering based upon on the 45,429 probes from the gene expression array (letters B, G, H, I, L, and M denote EIUM; O, P, Q, R, U, and X, denote OSIS). (C) The morphological features of EIUM and OSIS with and without IVD are shown using H&E (left 4 panels) and by immunofluorescence for ACTA2 counterstained with DAPI (right 4 panels). Scale bars represent 50 µm. Based on the array, differentially expressed genes were identified between (D) healthy and diseased cells, matched for IVD treatment and also as (E) a consequence of IVD for each cell type. The Venn diagrams show the total numbers of up- and downregulated genes identified in each comparison. A signature panel of key genes identified from these comparisons was validated by qPCR in the panels on the right. The disease status was the main effect impacting gene expression of *ESR1*, *PGR*, *NR5A1*, *ALDH1A2*, *MMP11*, and *HOXC6* (connecting bars; *p*<0.05; 2-way ANOVA). A significant interaction was observed between disease status and IVD treatment for ESR2, *CYP19A1*, *FOXO1*, *HAND2*, *PRL*, and *IGFBP1* (lower case letters indicate significantly different groups; *p*<0.05; Tukey's).

After normalization, 45,429 probes from the gene expression array were retained for analysis. Using principal component analysis (PCA), we examined the gene expression differences between 6 EIUM samples (B, G, H, I, L, M) and 6 OSIS samples (O, P, Q, R, U, X) both with (+) and without (−) *in vitro* decidualization (IVD) treatment. Nearly 66% of the variation across 45,429 probes was accounted for in the first two principal components (45.7% for PC1, 19.9% for PC2), and the samples clustered by both disease status and treatment when projected across these components (Figure 1A). Hierarchical clustering of the same data, shown in the dendrogram in Figure 1B, separated the samples into two primary groups based on disease status. Within these groups, samples were further divided primarily by treatment, although EIUM I- and OSIS O+ were exceptions to this second level of clustering.

Based on total signal from the array, one of the most abundant mRNAs in both cell types was smooth muscle actin (*ACTA2*). This was intriguing as we anticipated *ACTA2* expression in OSIS, but not EIUM. We saw this more vividly at the protein level (Figure 1C), where abundant ACTA2 fibers present in OSIS, both before and after IVD, coincided with some of the morphological differences seen in the diseased cells. ACTA2 protein was noticeably absent in EIUM, which typically appear as highly elongated, spindle-shaped cells that adopt a more rounded, epithelioid shape in response to IVD. OSIS are often elongated as well, but display a more irregular shape with a jagged or ruffled appearance along their edges. Moreover, OSIS cells were less rounded after IVD relative to EIUM.

We compiled lists of differentially expressed genes for later comparison to the methylation array, and to confirm the phenotype of the cells. Comparing OSIS to EIUM (Venn diagram Figure 1D), we identified 2,430 genes that were differentially expressed between the untreated groups, and 4,764 genes that were differentially expressed between the treated groups. Within each of these comparisons, the numbers of up- and downregulated genes were similar (i.e., in OSIS- vs. EIUM-, there were 1,143 upregulated genes and 1,287 downregulated genes). When the effect of treatment was examined in each population (i.e., EIUM+ vs. EIUM− and OSIS+ vs. OSIS−; Venn diagram Figure 1E), EIUM were observed to be more sensitive to IVD, with nearly 3 times as many genes differentially expressed in EIUM (4,549) compared with OSIS (1,517). Consequently, EIUM showed a large number of unique genes that changed with treatment (3,695) compared to OSIS (663). This suggests that the large differences in gene expression seen between normal and diseased cells are expanded further in response to IVD, when healthy stromal cells begin to decidualize but diseased cells showed a blunted response (for full gene lists from these comparisons please see Table S1).

Using qPCR, we validated a panel of genes that were differentially expressed on the array and representative of gene targets known to be differentially expressed in endometriosis or in response to IVD (right bar graph panels, Figures 1D, 1E). Consistent with previous reports, the expression of estrogen receptor alpha (*ESR1*), progesterone receptor (*PGR*), matrix metalloproteinase-11 (*MMP11*), and retinaldehyde dehydrogenase 2 (*ALD1A2*) was significantly lower in OSIS relative to EIUM; in contrast, the expression of estrogen receptor beta (*ESR2*), steroidogenic factor 1 (*NR5A1*), homeobox C6 (*HOXC6*), and aromatase (*CYP19A1*) was significantly higher in OSIS relative to the EIUM (*p*<0.05, main effect two-way ANOVA) [3], [23], [24]. Interestingly, both *ESR2* and *CYP19A1* showed a significant interaction between treatment and disease, with IVD increasing their expression in OSIS to a greater extent than in the other groups (*p*<0.001, Tukey's). The greatest differences in expression following IVD were seen for genes known to be induced during decidualization *in vivo*. The forkhead box protein O1 (*FOXO1*) as well as the heart and neural crest derivatives-expressed protein 2 (*HAND2*) are both essential mediators of the decidual response, and are increased in response to progesterone [22], [25]. Additionally, the well-characterized decidual markers prolactin (*PRL*) and insulin-like growth factor-binding protein 1 (*IGFBP1*) are strongly upregulated by IVD in EIUM but not OSIS [11]. There was a significant interaction across the groups for all 4 genes (*p*<0.05). In addition to confirming the results of the array, these differences function like a molecular signature highlighting established markers for both healthy and diseased cells and how they respond to IVD.

### Methylation differences define disease status

**Figure 2:**
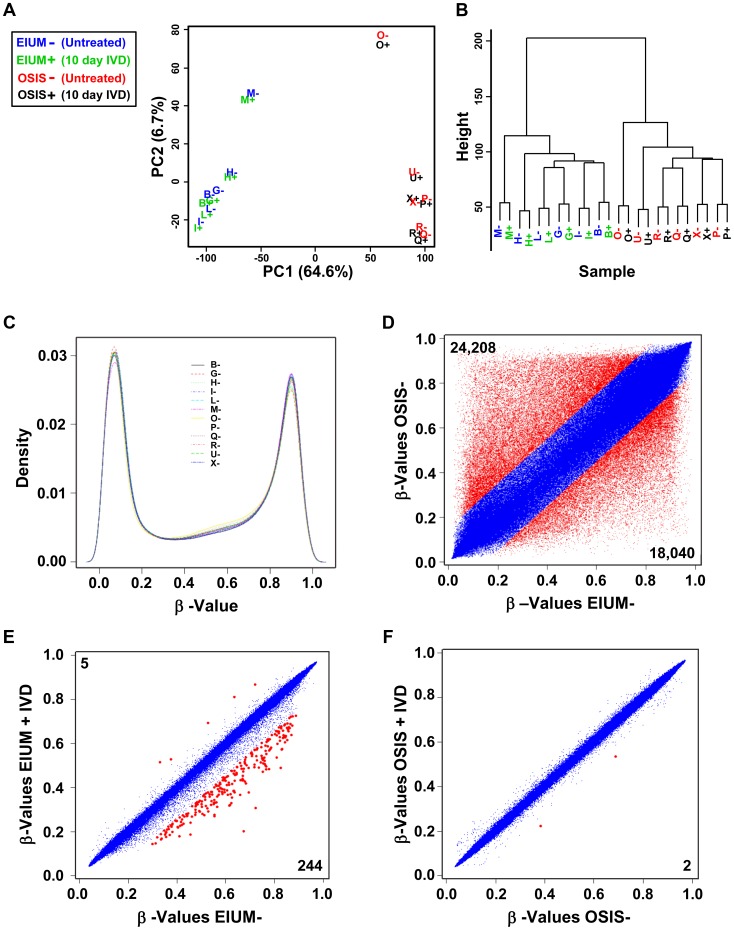
The global CpG methylation pattern in healthy and endometriotic stroma. The methylation in EIUM (n = 6) and OSIS (n = 6) with (+) or without (−) IVD was examined by (A) principal component analysis and (B) hierarchical clustering based upon on the 470,540 probes from the methylation array (letters B, G, H, I, L, and M denote EIUM; O, P, Q, R, U, and X, denote OSIS). The normalized density estimation was determined (C) from the β-values of all 12 untreated samples. Scatter plots compare the β-values for individual CpGs between (D) EIUM- and OSIS-, (E) EIUM+ and EIUM-, and (F) OSIS+ and OSIS−. Data plotted in red differed in average β-value by at least 0.15. Numbers in the upper left and lower right corners of each plot show the total number of hyper- and hypomethylated CpGs, respectively.

After normalization, 470,540 probes from the methylation array were retained for analysis. PCA of the methylation variation for all 12 samples, with and without IVD treatment, is shown in Figure 2A. More than 64% of the variation across all probes could be accounted for by the first principal component, and samples strongly clustered along this component based on their disease status (PC2 accounted for 6.7% of the variation). IVD treatment had little effect on the sample variation, and hierarchical clustering (Figure 2B) showed that subsequent branching was dictated by inter-sample variation. Density estimations derived from the full range of normalized β-values from each sample (Figure 2C) showed a similar bimodal frequency distribution of methylation. This indicates that global levels of methylation are comparable on somatic chromosomes, and that there is neither overall unidirectional shift in methylation nor substantial hemimethylation in either population.

We then examined how average β-values for individual CpGs on the array differed between groups. Untreated OSIS compared with untreated EIUM revealed the largest difference in methylation, with 42,248 differentially methylated CpGs. To visualize the differences in methylation at discrete CpGs, scatter plots were generated using average β-values from each group for each of the 470,540 CpGs (Figure 2D, β-values considered different shown in red, unchanged are in blue). For this comparison, there was a slightly higher number of probes showing greater methylation (24,208 CpGs, or approximately 57.3%) in OSIS relative to EIUM. Consistent with the PCA, only 249 CpGs were differentially methylated as a consequence of IVD treatment in EIUM (Figure 2E, red), and 244 of these showed a decrease in methylation. Only 2 CpGs were differentially methylated in OSIS with IVD treatment (Figure 2F, red; full lists of differentially methylated CpGs are provided in Table S2).

**Figure 3:**
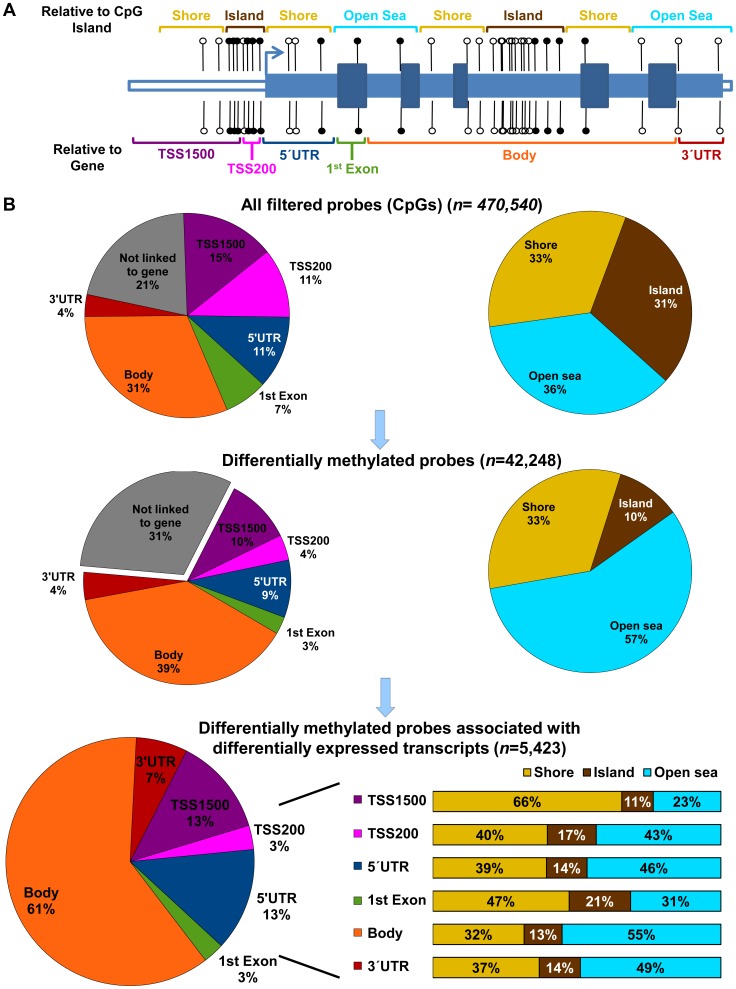
Genomic context of CpG methylation. A schematic diagram of CpGs (A) depicts their genomic context relative to the nearest CpG island (top) or gene (bottom). Island context was defined similarly to the Illumina manifest (see material and methods) as being within an “island” (brown), in a 4kb “shore” flanking the island (yellow), or in an “open sea” more than 4kb from an island. Gene context was defined relative to the nearest open reading frame being: within 1500 (TSS1500; purple) or 200 bp (TSS200; pink) of a transcription start site; in the 5′UTR (green), the 1^st^ exon of a transcript (green); in the body of gene (orange) or the 3′UTR (red). Pie charts (B) show the distribution of the CpGs examined based upon their genomic context for all probes retained from the array (top), for all the probes identified as differentially methylated between OSIS- and EIUM- (middle), and for all the differentially methylated probes that were matched to differentially expressed transcripts (bottom; note that stacked bar graphs show the CpG island context broken down for each of the gene contexts.).

The probe selection on the HumanMethylation450 beadchip is focused on genes and CpG islands (CGI). Accordingly, the annotations for individual CpGs on the array are subdivided based on their positional context relative to both nearby transcripts and the closest CGI (Figure 3A). Nearly 80% of the 470,540 CpGs examined on the array are linked to a transcript, mapping either near promoters (a range that includes upstream proximal promoters, the 5′UTR of transcripts, and the first exon of the gene), within the gene body (typically intronic), or to the 3′UTR of the transcript (Figure 3B, top pie charts). The annotation of the array's probes based on proximity to CGIs maps nearly one third of the CpGs within predefined CGIs, while another third map to regions flanking the CGIs, termed “shores” and defined as within 4 kb of the nearest island (as mentioned in the data analysis section, we merged Illumina's “shelf” and “shore” categories in to a single category, which we referred to as “shores”). CpGs unrelated to an island (i.e., more than 4 kb removed) comprised the remaining third, and were termed “open sea.”

We examined the gene context of the 42,248 differentially methylated CpGs that were identified between OSIS and EIUM, and found a similar breakdown to the full array; however, CpGs mapping near promoters (pink, purple, and blue) were slightly underrepresented relative to CpGs found in the body (orange) of transcripts (Figure 3B, middle). The CGI context showed a larger difference relative to the ratios built into the array. The majority of CpGs with differential methylation mapped to open seas (light blue), while the fraction of CpGs in islands (brown) was reduced to one third of what we expected from the array. We also noticed that only 2 of the CpGs identified as different before and after IVD mapped to a CpG island; however, we did not follow this group of CpGs in subsequent analyses because so few were differentially methylated (these are not represented graphically, see Table S2).

We then merged our results for the untreated OSIS and EIUM groups from the two arrays, and identified 1,402 differentially expressed mRNAs to which we could map 5,423 differentially methylated CpGs. We referred to these as matched CpGs (Table S2). Similar to above, matched CpGs occurred most frequently in the body of genes and distal to CGIs in open seas (Figure 3B, bottom). However, the combined breakdown revealed that matched CpGs found near gene promoters were more often associated with shores or islands, fitting with the convention that CGIs are more frequently found in these regions. Matched CpGs in the body of a gene were more often observed in open sea. Inspection of the matched CpGs in gene bodies revealed that 88% were distributed within introns; however, these intronic regions did not appear to preferentially overlap with known enhancers (data not shown).

**Figure 4:**
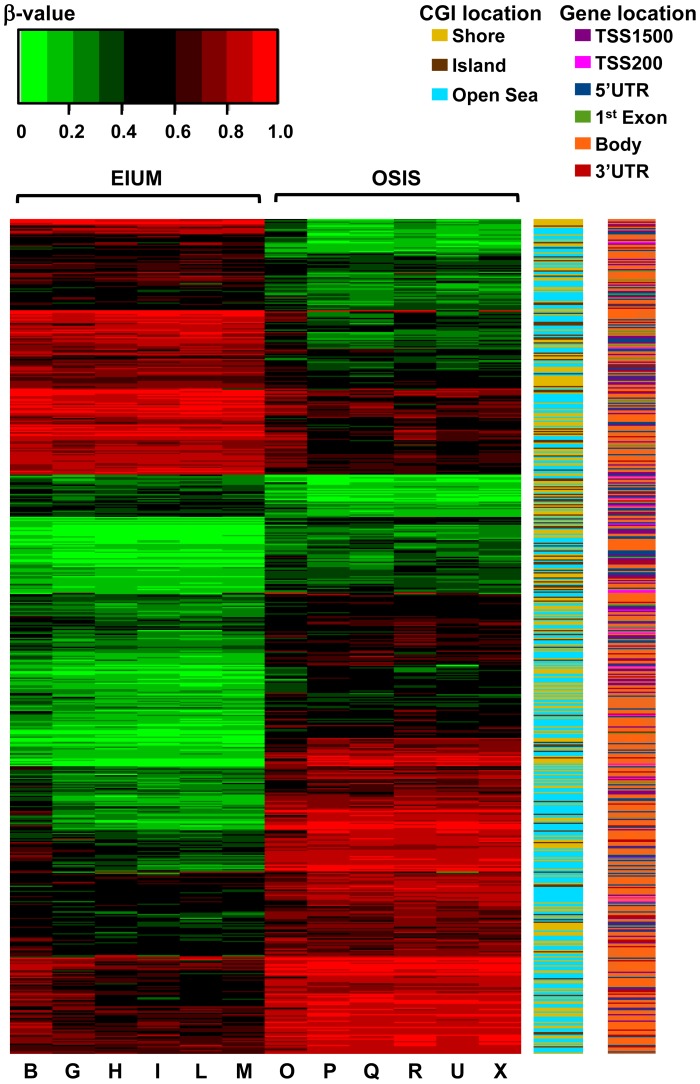
A combined heat map shows the differential methylation of the 5,423 matched CpGs in conjunction with gene and CGI context for EIUM and OSIS. Relative DNA methylation from each subject is encoded for each CpG from unmethylated (β-value = 0; green) to methylated (β-value = 1; red), and clustered based on the change in methylation. CGI and gene context are encoded in the right columns.

Because of the diverse distribution of these CpGs across different genomic contexts, we examined how changes in methylation were distributed across the matched genes based on CpG context. The heat maps in Figure 4 depict the direction of change in methylation between EIUM and OSIS side-by-side with the gene context and CGI context. Hypomethylated CpGs in OSIS are clustered near the top, whereas hypermethylated CpGs in OSIS are clustered near the bottom. The larger number of CpGs in gene bodies (orange) and in open seas relative to CGIs (light blue) is again apparent, but appears enriched near the bottom, suggesting they are more frequently hypermethylated. Additionally, a cluster of CpGs mapping to CGIs near promoters is observed more centrally, suggesting that the differences here are less pronounced.

Methylation is conventionally thought to repress gene expression; thus, we examined whether hyper- or hypomethylation at each of the matched CpGs correlated positively or negatively with gene expression (Table 1). When comparing the 5,423 differentially methylated and matched CpGs in OSIS and EIUM, nearly 64% of the CpGs showed hypermethylation (3,485). Interestingly, twice as many of the hypermethylated CpGs were negatively correlated with gene expression (2,363 vs. 1122). The 1,938 CpGs that were hypomethylated were more evenly divided between the positively and negatively correlated groups. We next constructed a test of proportions to examine the distribution proportion of positive and negative correlation at each CpG context. When stratified by gene context (Table 2), the number of differentially methylated CpGs in the first exon of a gene were more frequently negatively correlated with gene expression (*p*<0.00001), whereas CpGs in the 3′UTR showed a relatively higher proportion that positively correlated with gene expression (*p* = 0.01584). When stratified by island context (Table 3), differentially methylated CpGs that mapped to CGIs were also more likely to be positively correlated with gene expression (*p*<0.00001)

**Table 1:** Correlation of methylation with gene expression for matched CpGs.

Matched CpGs	total	positively correlated	negatively correlated to	ratio neg/pos
**Altered CpGs matched to an mRNA**	5423	2026	3397	1.68
**Matched hypomethylated CpGs**	1938	904	1034	1.14
**Matched hypermethylated CpGs**	3485	1122	2363	2.11

The differentially methylated CpGs that matched to differentially expressed genes are sorted above based on the direction with which gene expression correlated with methylation. A positive correlation indicates that hypermethylation matched with upregulation or hypomethylation matched with gene repression. A negative correlation indicates that hypermethylation matched to a repressed gene or that hypomethylation matched to an unregulated gene. The last column shows the ratio of negatively to positively correlated CpGs for each subgroup in the first column.

**Table 2:** Correlation of methylation with gene expression based on gene context.

Matched CpGs by Gene Context	total	positively correlated	negatively correlated	ratio neg/pos	*p*-value
**TSS1500**	690	275	415	1.51	0.15418
**TSS200**	171	57	114	2.00	0.29707
**5′UTR**	726	259	467	1.80	0.28532
**1stExon**	149	22	127	5.77	<0.00001
**Body**	3327	1256	2071	1.65	0.43776
**3′UTR**	360	157	203	1.29	0.01584

The matched CpGs are sorted by their genomic context and then based upon the direction with which gene expression correlated with methylation. The *p*-value comes from a test of proportions on the Spearman rank correlation coefficient obtained for the ratios of positively and negatively correlated CpGs at each location.

**Table 3:** Correlation of methylation with gene expression based on island context.

Matched CpGs by CGI context	total	positively correlated	negatively correlated	ratio neg/pos	*p*-value
**Island**	724	325	399	1.23	0.00002
**Shore**	2060	689	1371	1.99	0.09809
**Open Sea**	2639	1012	1627	1.61	<0.00001

The matched CpGs are sorted by their island context and then based upon the direction with which gene expression correlated with methylation. The *p*-value comes from a test of proportions on the Spearman rank correlation coefficient obtained for the ratios of positively and negatively correlated CpGs at each location.

### ANOVA interaction modeling of differentially methylated and differentially expressed genes

We wanted to statistically infer the impact of methylation across a given gene on its expression. While several statistical approaches have been used to report average weighted changes in methylation across a given gene or region of chromatin, the large and punctuated variations in methylation we observed across the many different contexts suggested that variable differences in methylation might be a more useful model for correlating methylation with gene expression. To examine this, we used ANOVA to evaluate methylation variation across each matched gene. This approach was based on the prediction that statistically significant interactions across multiple gene or CGI contexts would identify genes more likely to have their expression affected by differential methylation. Consequently, cross-group interactions would preferentially identify genes where the methylation differences were both highly different in β-value and also widely distributed across unique contexts within the gene. At the same time, genes without multiple differentially methylated CpGs, or genes where methylation differences are clustered together, would be devalued. The ANOVA identified 403 genes (Table S1) with a statistically significant interaction (adjusted *p*<0.05) among their CpGs. This represented 2,978 of the differentially methylated CpGs (Table S2). One remarkable finding from this analysis was that it correctly identified the *HOXA* cluster, *NR5A1*, and *PGR*—genes that are aberrantly methylated and differentially expressed in endometriosis—as highly significant. The 403 genes identified by ANOVA were uploaded to MetaCore and classified by protein function using ORA (Table 4). While many classes were present (kinases, receptors, etc.), transcription factors were the only functional class to reach statistical significance (*p* = 4.01×10^−9^).

**Table 4:** Enrichment by protein function of ANOVA-identified genes.

Protein class	Actual	R	Expected	Ratio	*p*-value	z-score	In data set	In protein function	Protein function in database
**Transcription factors**	45	959	17.13	2.63	4.02E-09	6.934	10.56%	4.69%	4.02%
**Phosphatases**	7	230	4.109	1.70	1.20E-01	1.446	1.64%	3.04%	0.96%
**Ligands**	13	514	9.183	1.42	1.34E-01	1.285	3.05%	2.53%	2.16%
**Kinases**	16	654	11.68	1.37	1.29E-01	1.292	3.76%	2.45%	2.74%
**Enzymes**	54	2724	48.67	1.11	2.26E-01	0.8195	12.68%	1.98%	11.42%
**Proteases**	11	559	9.987	1.10	4.15E-01	0.3272	2.58%	1.97%	2.34%
**Receptors**	30	1565	27.96	1.07	3.71E-01	0.4026	7.04%	1.92%	6.56%
**Other**	253	16691	298.2	0.84	1.68E-06	-4.822	59.39%	1.52%	70.00%

Over representation analysis of the 403 ANOVA interaction-identified genes was performed based on broadly defined protein functions. The Metacore database for the human genome annotates 23,844 network objects for the background list. Column abbreviations: *Actual*, number of ANOVA identified objects for a given class; *R*, number of background objects for a given class; *Expected*, mean value for hypergeometric distribution (403*R/23,844); *Ratio*, the ratio of actual/expected; *p-value*, probability to have the given actual value or higher (or lower for negative z-score); *In data set*, percent of objects of a given function in the ANOVA dataset; *In protein function*, percent of ANOVA objects of a given function relative to the number of background objects with the same function; *Protein function in database*, percentage of ANOVA objects of a given function in the background list.

**Figure 5:**
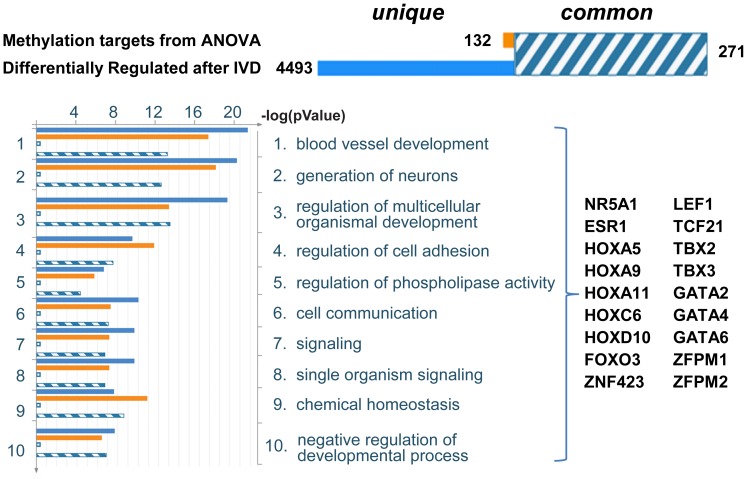
Summary of statistical comparison between ANOVA identified genes and genes differentially expressed after IVD. ANOVA interaction identified 403 genes both differentially methylated and differentially expressed between OSIS- and EIUM-. The gene expression array identified 4,764 differentially expressed transcripts between OSIS+ and EIUM+. The intersection of these two gene lists identified 271 common genes (shown at the top in hashed blue and white). In the same fashion, we examined the gene ontology cellular process terms that were similar between the two groups, to look for differentially methylated genes and transcription factors that may serve as pathway hubs during IVD. The most overrepresented terms were determined for the both groups of genes. The intersection of similar GO terms was then sorted based on *p*-value (bottom left), and the gene symbols for transcription factors common to both groups that contributed to this list of GO terms are listed (bottom right).

To identify differentially methylated genes that may potentially alter the pathways involved in decidualization, we first identified the enriched GO processes among the differentially expressed genes observed when comparing IVD-treated OSIS and EIUM (4,764 transcripts shown in Figure 1C). We then determined which of these groups had significant overlap with the genes we identified by ANOVA interaction. The top processes identified through this intersection are shown in Figure 5, and included several pathways important for the development and progression of endometriosis, such as organ development (patterning), blood vessel development, neuronal development, and regulation of cell adhesion. From this we identified subsets of differentially methylated genes that encoded transcription factors enriched as hubs within these processes.

### Genes encoding key transcription factors are differentially methylated in endometriosis

**Figure 6:**
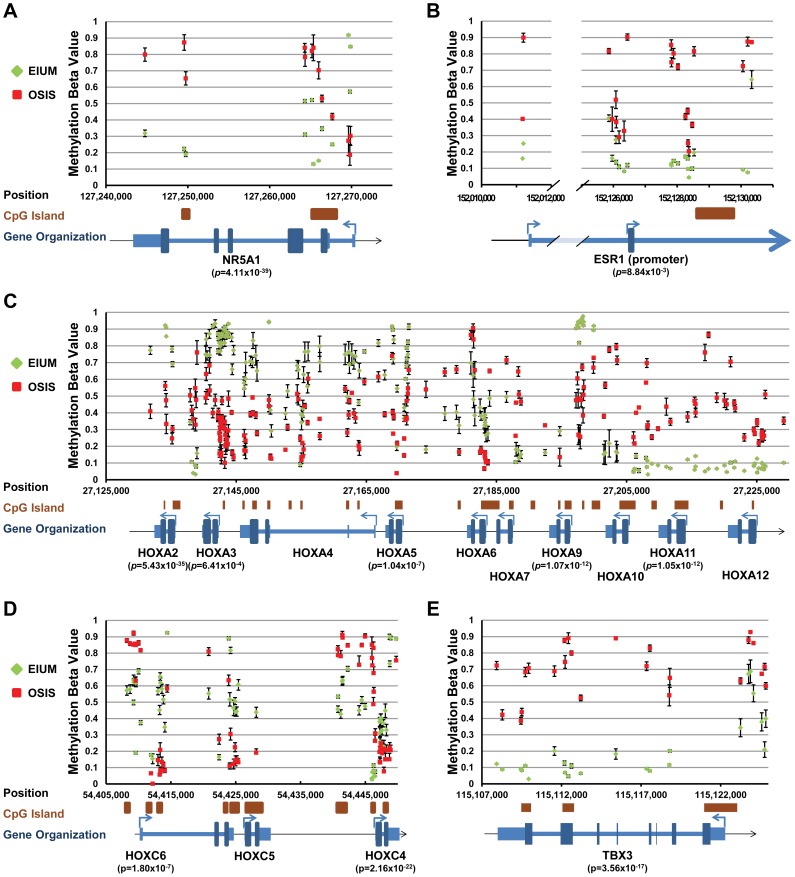
Key transcription factors with differential methylation (part 1). Methylation maps for the following five genes/genomic regions show the differentially methylated CpGs identified by ANOVA interaction analysis: (A) *NR5A1*; (B) *ESR1* promoter regions; (C) portion of the *HOXA* cluster; (D) portion of the *HOXC* cluster; (E) *TBX3*. Each plot shows the average β-value for untreated EIUM (green diamond) and OSIS (red squares) at the genomic coordinate for each identified CpG (error bars show SEM). CGI's and gene diagrams are illustrated below each plot. (When significant, the lowest *p*-value obtained from the ANOVA interaction is shown below gene symbols.)

**Figure 7:**
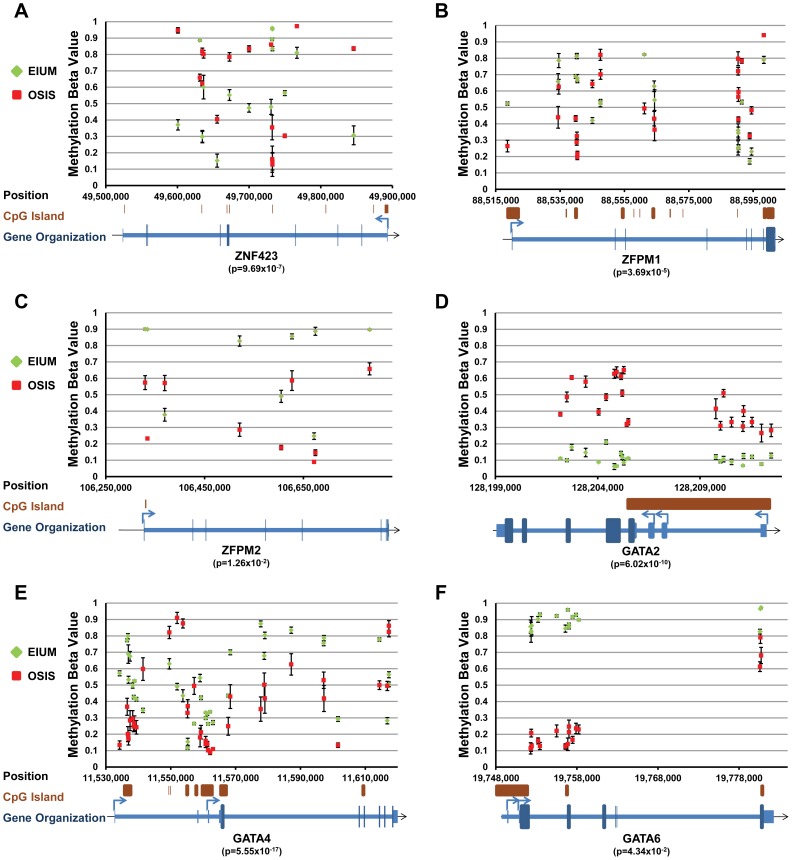
Key transcription factors with differential methylation (part 2). Methylation maps for the following six genes show the differentially methylated CpGs identified by ANOVA interaction analysis: (A) *ZNF423*; (B)*ZFPM1*; (C) *ZFPM2*; (D) *GATA2*; (E) *GATA4*; (F) *GATA6*. Each plot shows the average β-value for untreated EIUM (green diamond) and OSIS (red squares) at the genomic coordinate for each identified CpG (error bars show SEM). CGI's and gene diagrams are illustrated below each plot. (When significant, the lowest *p*-value obtained from the ANOVA interaction is shown below gene symbols.)

We examined the methylation profiles at 11 regions that we predicted to be important in controlling the expression of transcription factors identified by ANOVA and in the enrichment analyses. Since these were identified by ANOVA, these genes were all both differentially methylated and differentially expressed on our arrays (Tables S1 and S2). The methylation plots for these regions were built by aligning the differentially methylated CpGs along diagrams for the associated genes (Figures 6 and 7; orientation is relative to the “+” strand). Additionally, the average β-values for these CpGs from either EIUM or OSIS are plotted along the y-axis. The methylation profile for *NR5A1* (*p* = 4.11×10^−39^, Figure 6A) matches with those of previous reports for these cell types: relative to EIUM, the *NR5A1* gene promoter in OSIS is hypomethylated but the gene body is hypermethylated [26], [27]. Notably, we identified several regions of hypermethylation in the *ESR1* gene (*p* = 8.84×10^−3^) near its 3′ promoter as well as in regions flanking an intronic CGI (Figure 6B). The *ESR1* gene has been extensively studied in EIUM and OSIS, but differences in its methylation status have not been previously reported. *HOXA10* and *HOXA11* are known to be differentially methylated in endometriosis [28], [29]. Our results extend these findings by identifying multiple regions across the *HOXA* cluster that are differentially methylated in OSIS relative to EIUM (*p*-values are listed for the *HOXA* genes identified by ANOVA; Figure 6C). A unique pattern of differential methylation was seen across the central portion of the *HOXC* cluster on chromosome 12, where both hypo- and hypermethylation occurred at CGI shores near the promoters of *HOXC4* (*p* = 2.16×10^−22^), *HOXC5*, and *HOXC6* (*p* = 1.80×10^−7^, Figure 6D). The role of the *HOXC* genes in the endometrium is unknown. *HOXC6* is regulated in part by two estrogen response elements in its promoter [30], and is highly expressed in ovarian endometriotic tissue relative to eutopic tissue [23]. We also observed high levels of *HOXC6* in OSIS relative to EIUM (qPCR panel in Figure 1C), and the array reported higher levels of *HOXC4* and *HOXC8* as well. The T-box transcription factor 3 (*TBX3*) is important for lineage decision and cell fate guidance during embryonic development, and the CGI overlapping its promoter and first exon is frequently differentially methylated in cancer [31], [32]. We saw *TBX3* expressed in EIUM but not in OSIS on our array, and noted that a CGI and much of this gene were uniformly methylated in OSIS (*p* = 3.56×10^−17^) (Figure 6E). In contrast, the zinc finger protein 423 (*ZNF423*) was an example where hypermethylation throughout the gene body in OSIS was positively correlated with expression on our arrays (Figure 7A).

One of the most striking differences identified in this analysis was the high representation of GATA transcription factors and transcriptional coregulators of the GATA family. The zinc finger protein genes *ZFPM1* and *ZFPM2*, often referred to as friends of GATA, showed unique patterns of mixed methylation. In EIUM the 5′ region of *ZFPM1* gene showed increased methylation while its 3′ region had reduced methylation. This pattern was reversed in OSIS where *ZFPM1* had reduced methylation at its 5′ end relative to the 3′ (Figure 7B). The ZFPM2 gene was largely hypomethylated intragenically in OSIS relative to EIUM, although the absolute differences in methylation varied across the gene (Figure 7C). Both ZFPM1 and ZFPM2 were upregulated in OSIS relative to EIUM). Three *GATA* isoforms showed altered methylation. Multiple CpGs throughout the promoter and body of *GATA2* showed higher methylation in OSIS relative to EIUM (*p* = 6.02×10^−10^, Figure 7D), while *GATA4* and *GATA6* had less methylation across ranges of intronic CpGs flanking CGIs (7E, *p* = 5.55×10^−17^ and 7F, *p* = 4.34×10^−2^). Similar to the HOX genes, the individual methylation status of these GATA members was inversely correlated with gene expression, but because GATA family members are encoded on separate chromosomes, the observed methylation differences are not a shared occurrence. Because little is known regarding the role of GATA family members in the endometrium and endometriosis, we examined these genes more closely.

### GATA isoform expression in EIUM and OSIS

**Figure 8:**
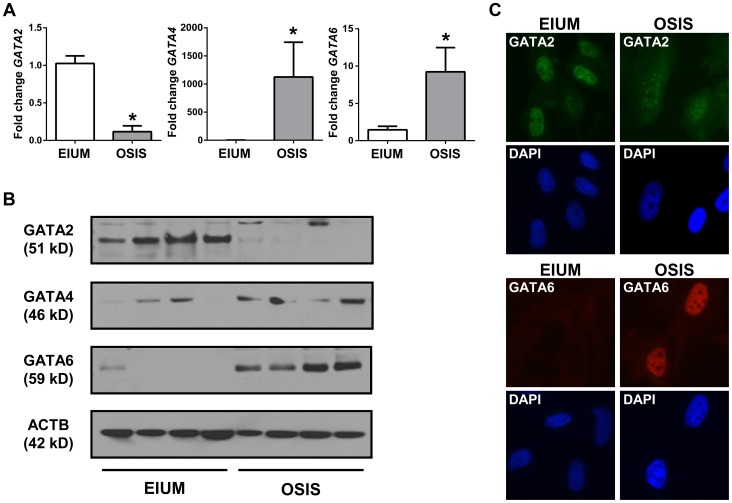
GATA isoform expression in stromal cells. The expression of GATA2, GATA4, and GATA6 in EIUM and OSIS is shown using (A) qPCR (asterisks indicate p<0.05, t-test, n = 6), (B) immunoblot for 4 EIUM and 4 OSIS samples, (C) immunofluorescence for GATA2 (pseudocolored green; top panels) and GATA6 (red; bottom panels) in EIUM (left column) and OSIS (right panel), using DAPI as a nuclear counterstain.

**Figure 9:**
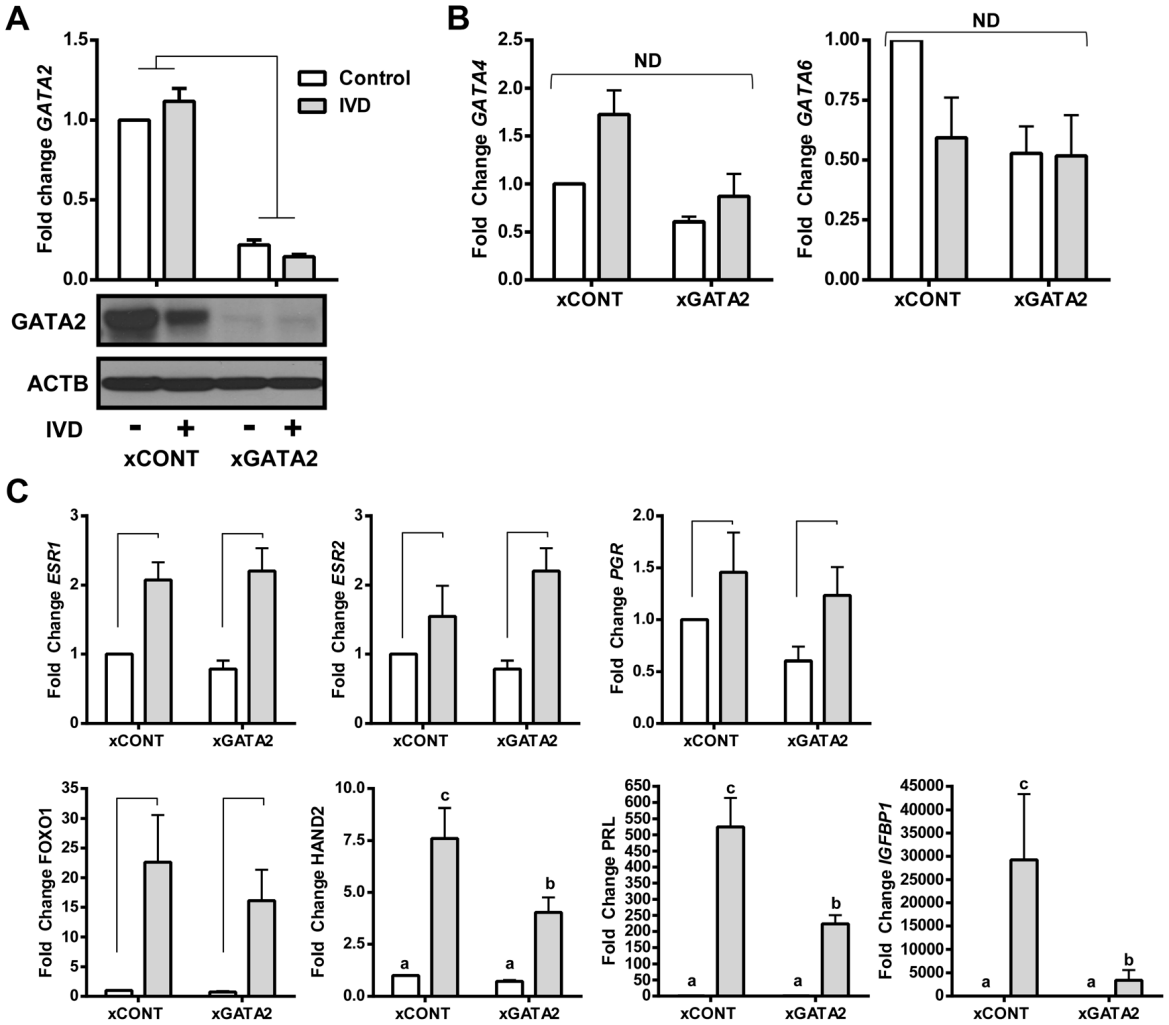
The effects of silencing GATA2 in EIUM. (A) Transfecting 50 nM of non-targeting control siRNA (xCONT) had little effect, but siRNAs targeting human GATA2 (xGATA2) effectively silenced *GATA2* mRNA (qPCR; top), and protein (immunoblot; bottom). (B) Neither siRNA transfection nor IVD treatment significantly affected the expression of *GATA4* or *GATA6* mRNA, as shown by qPCR. (C) qPCR showed IVD was the main effect for altered expression of the steroid hormone nuclear receptors *ESR1*, *ESR2*, *PGR*, as well as *FOXO1*. However, the IVD-induced expression of decidualization markers *HAND2*, *PRL*, and *IGFBP1* was significantly reduced in response to silencing GATA2. Other markers from our panel showed no change with any treatment (not shown). (n = 5; connecting bars show main effect *p*<0.05 by 2-way ANOVA; lower case letters indicate significantly different groups *p*<0.05 by Tukey's after ANOVA).

**Figure 10:**
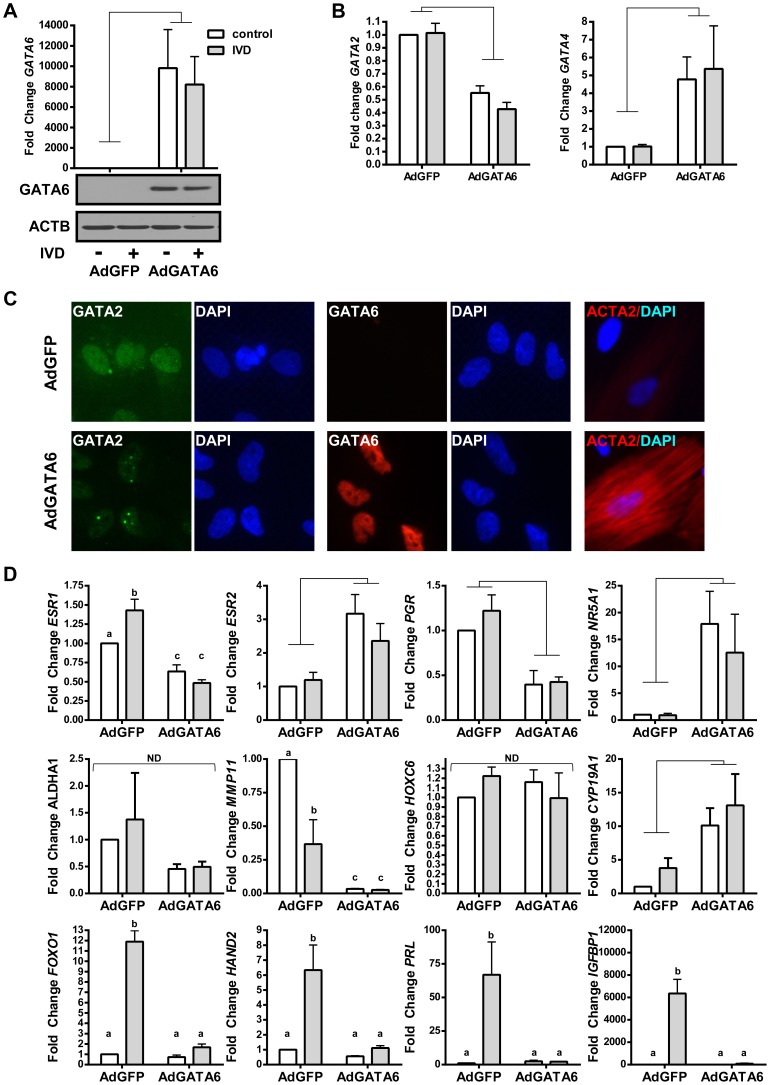
The effects of GATA6 expression in EIUM. (A) Adenoviral-mediated expression of GATA6 (AdGATA6) resulted in significant mRNA (top) and immunoreactive protein (bottom) relative to cells transduced with an adenovirus expressing GFP (AdGFP). IVD did not significantly affect GATA6 expression. (B) qPCR showed that GATA6 transduction was the main affect for the repression of GATA2 and the induction GATA4 mRNA. (C) Immunofluorescence for GATA2 (pseudocolored green), GATA6 (red; middle panels), and ACTA2 (red; right panels) is shown for AdGFP-transduced cells (top row) and AdGATA6-transduced cells (bottom row). Transcription factors showed proper nuclear staining, and GATA2 nuclear signal was reduced when GATA6 was present (DAPI counterstaining is shown beside panels for transcription factors). ACTA2 immunoreactivity was observed in AdGATA6 transduced cells similar to OSIS (compare to figure 1; DAPI counterstain is merged). (D) qPCR was performed on the same panel of 12 genes shown in Figure 1, and EIUM cells transduced with AdGATA6 resulted in a shift in gene expression that resembled OSIS. AdGATA6 was the main effect for the observed change in *ESR2*, *PGR*, *NR5A1*, and *CYP19A1*. A significant interaction was observed between AdGATA6 treatment and IVD for *ESR1*, *MMP11*, *FOXO1*, *HAND2*, *PRL*, and *IGFBP*. *HOXC6* and *ALDH1A2* were not significantly affected by either treatment. (n = 4; connecting bars show main effect *p*<0.05 by 2-way ANOVA; lower case letters indicate significantly different groups p<0.05 by Tukey's after ANOVA).

**Figure 11:**
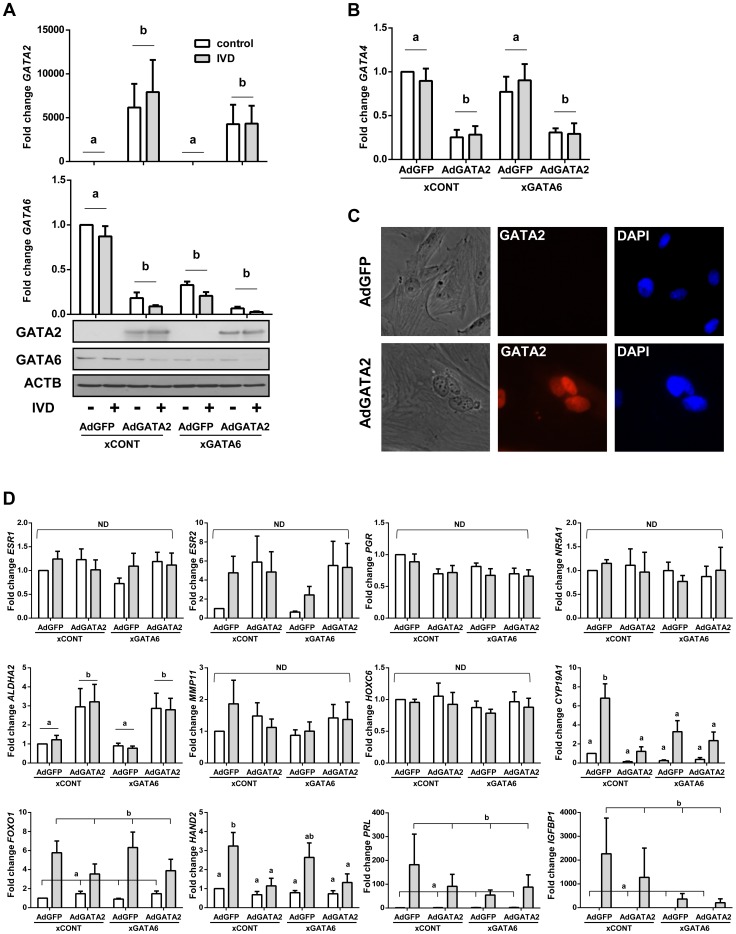
The effect of silencing *GATA6* and expressing GATA2 in OSIS. (A) Manipulating GATA expression in OSIS was accomplished using adenoviral transduction to express GATA2 (AdGATA2) and siRNAs targeting human *GATA6* (xGATA6). Transduction resulted in significant mRNA (top) and immunoreactive GATA2 (bottom) relative to cells transduced with an adenovirus expressing GFP. Compared to control siRNAs, xGATA6 effectively silenced *GATA6* mRNA (qPCR; middle), and protein (immunoblot; bottom). IVD did not significantly affect the expression of any of the GATAs examined, but AdGATA2 strongly repressed (A) *GATA6* and (B) *GATA4*. (C) Phase contrast (left), immunofluorescence for GATA2 (red), and DAPI staining (right) shows nuclear GATA2 protein in AdGATA2-transduced cells. (C) qPCR was performed on the same panel of 12 genes shown in Figure 1. AdGATA2 was the main effect for the observed increase in *ALDH1A2*, while xGATA6 and AdGATA2 both repressed *CYP19A1*. IVD was the main effect for *FOXO1*, *HAND2*, *PRL*, and *IGFBP*. The *ESR1, ESR2, PGR, NR5A1*, *HOXC6* and *MMP11* were not significantly affected by any of the treatments. (n = 6; connecting bars show main effect *p*<0.05 by 2-way ANOVA; lower case letters indicate significantly different groups *p*<0.05 by Tukey's after ANOVA).

Our microarray analysis demonstrated that *GATA2* was more abundant in EIUM, whereas *GATA4*, *GATA6*, *ZFPM1*, and *ZFPM2* were more abundant in OSIS. To validate this, we examined mRNA and protein expression for all 5 genes in both cell types. *GATA2* mRNA was 8.7-fold lower in OSIS than EIUM (Figure 8A), whereas *GATA4* and *GATA6* were 1100-fold higher and 9.2-fold higher, respectively, in OSIS. Importantly, the qPCR results suggested that the abundance of *GATA4* in EIUM and OSIS (C_T_ values from 35 to undetectable) was very low relative to *GATA2* and *GATA6* (C_T_ values around 27)—thus although the fold induction of *GATA4* was the largest, it was abundance was scarce relative to both the other isoforms. The data for the ZFPMs was less clear-cut. The mRNA levels of *ZFPM1* and *ZFPM2* were 1.8-fold and 2.4-fold higher (respectively) in OSIS than in EIUM (Figure S1). Only ZFPM2 was detectable at the protein level, where it was consistently expressed in OSIS, but more variably in EIUM (ZFPM1 was undetectable using two different commercial antibodies). Immunoblots showed that GATA2 was highly abundant in EIUM, but scarcely detectable in OSIS (Figure 8B). Immunofluorescence for GATA2 (Figure 8C) confirmed strong, uniform nuclear signal in EIUM, but OSIS cells stained with a weaker, more diffuse signal with a unique punctate appearance. GATA4 was detectable in both EIUM and OSIS by immunoblot, with slightly variable expression, but no signal was observed in either cell type by immunofluorescence. Immunoblots and immunofluorescence showed that GATA6 was robustly expressed and localized to the nuclei in all OSIS samples but barely detectable in EIUM. (Notably, IVD had little effect on the expression of any of the GATA family members examined–compare figure 9A & 10A for EIUM, Figure 11A for OSIS.)

Given the striking differences in *GATA2* and *GATA6* expression, we proceeded to more closely examine their expression and function. We first validated the methylation data from the array using methylation specific PCR (Figure S2). Primers mapping to either exon 4 of *GATA2* showed that this region was fully unmethylated in EIUM, and was predominantly methylated in OSIS. Primers targeting exon 2 of *GATA6* showed that it was fully methylated in EIUM and fully unmethylated in OSIS.

### Depleting GATA2 reduces the decidual response of EIUM

Given the stark differences in GATA expression in EIUM and OSIS, and that endogenous GATA2 was present only in EIUM, we wanted to examine how GATA2 affected gene expression and the decidual response in EIUM. We transiently transfected EIUM with gene-specific siRNAs (xGATA2, Figure 9) or scrambled siRNA controls (xCONT), and the cells underwent IVD or control treatment for 6 days. *GATA2* mRNA and protein were reduced in EIUM by more than 80% (Figure 9A); however, the loss of GATA2 did not affect the transcription of *GATA4* or *GATA6* (Figure 9B). Similarly, the expression of the nuclear steroid hormone receptors were not significantly affected by the loss of GATA2 (Figure 9C), nor were the other genes from our panel whose expression varied only as a function of disease (*NR5A1*, *HOXC6*, *CYP19A1*, and *ALDH1A2* (not shown). In contrast, when we examined the array of genes known to be differentially expressed in response to IVD (shown in Figure 1) we found that silencing GATA2 significantly reduced the established markers of decidualization (Figure 9C, bottom row). In the absence of GATA2, the induction of *HAND2* and *PRL* in response to IVD was reduced by 47% and 57%, respectively, and IGFBP1 induction was reduced by 88% (*p*<0.05; Tukey's). The expression of FOXO1 was slightly reduced, but was not significant. This suggested that GATA2 expression may enhance decidual response.

### GATA6 expression in EIUM induces markers of endometriosis

Using adenoviral vectors, we introduced either eGFP (AdGFP) or human GATA6 (AdGATA6) into EIUM, treated the cells for 6 days with or without IVD, and then followed the effects of GATA6 on our panel of differentially expressed genes. The adenovirus significantly increased expression of the recombinant gene, and GATA6 expression was still abundant after the 6-day culture (Figure 10A). As before, IVD did not affect GATA6 or the other GATA family members (Figure 10B), but the overexpression of GATA6 resulted in a 2-fold reduction in *GATA2* mRNA (p<0.001) and a 4-fold increase in *GATA4* mRNA (p<0.001). The effect of GATA6 overexpression on GATA2 protein levels was more pronounced (Figure 10C), with expression decreased and redistributed in the nuclei to produce a punctate pattern similar to what we observed in OSIS in Figure 8C (GATA2 pseudocolored from red to green due the use of GFP; GATA6 in red, Figure 10C). GATA6 overexpression also appeared to affect the cytoskeleton of EIUM, with a potent increase in ACTA2 expression that again resembled what we saw in OSIS (see Figure 1E).

The qPCR panel in EIUM cells overexpressing GATA6 revealed a pronounced shift in gene expression that mirrored the expression patterns we saw in OSIS (compare Figure 10D and Figure 1 qPCR). All 4 of the nuclear receptors that we examined showed significant changes in expression. *ESR1* mRNA was reduced by 1.6-fold when GATA6 was overexpressed without IVD, and by 3-fold after IVD (both *p*<0.05). The overexpression of GATA6 also reduced *PGR* transcript levels an average of 2.6-fold (*p*<0.01). In contrast, but consistent with the OSIS qPCR results in Figure 1, the expression of *ESR2* and *NR5A1* were increased an average of 2.5-fold (*p*<0.05) and 22.4-fold (*p*<0.05), respectively, and IVD did not significantly affect these genes. *MMP11* and *CYP19A1* mRNA levels were also strikingly altered by GATA6 overexpression. *MMP11* was repressed by IVD (by 2.7-fold), but overexpression of GATA6 further reduced its transcript levels by 30-fold (*p*<0.01), relative to untreated controls. As before, *CYP19A1* was expressed at very low levels basally, but this was significantly increased after GATA6 overexpression (8.5-fold without IVD, 13.3-fold with IVD, *p*<0.01). *HOXC6* and *ALDH1A2* did not change following either IVD or GATA6 overexpression. Finally, overexpression of GATA6 profoundly restricted the ability of EIUM to decidualize, with all four of the genes expected to increase with IVD (*FOXO1*, *HAND2*, *PRL*, and *IGFBP1*) effectively blocked by GATA6 (*p*<0.01).

### Restoring GATA expression in OSIS does not rescue hormone response

The striking effects of altering GATA expression in EIUM led us to hypothesize that restoring the profile of GATA family members in OSIS to that seen in EIUM might recover the decidual response. Our experiments in EIUM showed that depleting GATA2 did not enhance GATA6 expression, and we anticipated that our experiments in OSIS would require simultaneous depletion of GATA6 and overexpression of GATA2. To this end we used siRNAs to knockdown *GATA6* in OSIS while simultaneously expressing GATA2 (AdGATA2) via adenoviral transduction. The xCONT scrambled siRNA and eGFP expressing adenovirus were again used as controls. This was done both in the presence or absence of IVD treatment for 6 days (n = 7).

Adenoviral-mediated expression of GATA2 increased mRNA and protein expression of GATA2 (Figure 11A). After 6 days of culture, the *GATA6* siRNAs reduced *GATA6* mRNA by 74%, and protein levels by 51%. Remarkably, the expression of GATA2 also potently blocked *GATA6* mRNA and protein, and the combined knockdown of GATA6 and overexpression of GATA2 reduced GATA6 mRNA by 95% and protein by 92% (Figure 11A). Intriguingly, *GATA4* mRNA expression in OSIS was reduced by GATA2 overexpression but not GATA6 knockdown (Figure 11B). Exogenously expressed GATA2 protein was observed to be completely nuclear (Figure 11C).

We were surprised to observe that only 2 genes in our panel were significantly affected in response to manipulating GATA expression: *ALDH1A2* and *CYP19A1* (Figure 11D). The nuclear hormone receptors were not affected by any treatments, although there was a trend for increased basal *ESR2* after GATA2 overexpression. Likewise, neither silencing endogenous GATA6 nor expression of GATA2 improved the decidual response in OSIS. Quite opposite, GATA2 overexpression appeared to block the slight induction of *HAND2* seen after IVD in OSIS, while PRL and IGFBP trended downward. As a result, IVD was the main effect for the expression of *FOXO1*, *HAND2*, *PRL*, and *IGFBP1* in OSIS, the relatively heterogeneous expression demonstrated that subject-to-subject variation was greatly affecting these genes. The expression of *ALDH1A2* was induced by GATA2 by 3-fold, but was not affected by IVD or GATA6 depletion. The expression of *CYP19A1* revealed a more complex expression pattern in response to these treatments. As before, IVD strongly increased *CYP19A1* expression. Silencing GATA6 decreased basal *CYP19A1* by 87%, although this did not reach statistical significance. Moreover, silencing GATA6 nearly blocked IVD-stimulated CYP19A1. Fitting with this, the expression of GATA2, and concomitant loss of GATA6, affected *CYP19A1* expression in a pattern similar to GATA6 depletion.

## Discussion

We found that endometriotic cells possess a unique epigenetic fingerprint compared to healthy endometrial stromal cells. Moreover, we identified a large network of transcriptional regulators differentially methylated in endometriosis and linked to decidualization. This included a surprising number of GATA family members. With further examination of GATA2 and GATA6, we found that GATA2 strongly regulates genes essential for decidualization, whereas GATA6 promotes an endometriotic phenotype. From this we suggest the possibility that an epigenetic switch controlling GATA isoform expression is important in the progression of endometriosis.

Focused efforts and better techniques are rapidly improving our understanding of how DNA methylation affects cell differentiation and human disease. The converging evidence that endometriosis is linked evolutionarily to decidualization, that steroid-dependent pathways are dysregulated in the disease, and that DNMTs are differentially expressed and regulated by steroid hormones in both cell types led us and many others us to speculate that both decidualization and endometriosis could be affected by dynamic epigenetic cues arising from DNA methylation [21], [33]–[38]. This appears to be the case in endometriosis, as the number of genes aberrantly methylated in endometriosis continues to grow, and the mutable and heritable nature of DNA methylation fits well as a mechanism to help to explain the enigmatic occurrence of endometriosis [3], [4], [39], [40]. Our data suggests that this epigenetic component is a defining feature of endometriosis, and also helps to unify many of the diverse observations regarding its origin. While it is our opinion that Sampson's model can explain most instances of endometriosis, it is clear that lesions can arise through other mechanisms such the induction of either ectopic mesenchymal cells or stem cells, or similarly by müllerianosis [5]. We suggest that epigenetic defects in the unique genetic pathways of the primate underlie all these mechanisms, and that the spontaneous occurrence of endometriosis in primates can be traced to an epigenetic plasticity in mesodermal mesenchymal cells.

Borghese et al. published the first global survey of DNA methylation in whole endometriotic tissues using MeDIP arrays to profile specific promoters [41]. Their work suggested that global patterns in methylation are similar between endometriosis, and that variation in methylation was more likely to occur at discreet loci across the genome. The gene-centric focus of our array compliments and expands these observations, and we observed genome-wide differences more frequently in the body of genes, and in the shore and open sea areas that flank CpG islands. Four of the loci they reported mapped to genes that we identified by ANOVA interaction: 5′-AMP-activated protein kinase subunit gamma-2 (*PRKAG2*), *HOXD10*, zinc finger protein 22 (*ZNF22*), and the anoctamin 1, calcium activated chloride channel (*ANO1*). In addition to having differentially methylated promoters, we saw methylation differences in the body and UTRs of these genes. These genes show a consistent correlation between DNA methylation and gene expression which is altered in endometriosis, although their function in the endometrium remains largely unknown. *HOXD10* has been implicated in endometrial stromal cell proliferation, and our pathway analysis comparison identified it as well, suggesting that all of the HOX clusters may show aberrant methylation in endometriosis [42].

Notably, we saw very few changes in DNA methylation in response to IVD treatment. When changes did occur, they were almost exclusively instances of hypomethylation. Interestingly, one of these in EIUM mapped to IGFBP1, which is massively upregulated in healthy cells both *in vivo* and *in vitro* in response to progesterone. It seems likely that the slightly increased number of CpGs that change in EIUM after IVD treatment reflects their increased sensitivity and dynamic response to steroid hormones, whereas the paucity of CpGs in OSIS that change with treatment underscore its more rigidly differentiated phenotype. Gao et al. recently used methylation-sensitive restriction fingerprinting to examine DNA methylation in the mouse endometrium during pregnancy and pseudopregnancy; they were able to identify several loci where differential methylation was induced during decidualization, although these did not correlate with our current findings [36].

It is important to recognize that the Gao and Borghese groups both used whole tissue as well as different platforms to interrogate DNA methylation, and these differences highlight important limitations when interpreting and comparing results. Our present study made extensive use of homogenous cultures of first passage primary stromal cell cultures, isolated several days in advance of starting the experiment. While the examination of whole tissue fragments taken fresh *ex vivo* is clearly relevant, and may ultimately be more informative, it is well established that large epigenetic differences occur between different cell and tissue types [43]. Since the Illumina beadchip can discern very subtle differences in methylation, and the signal from heterogeneous samples can deteriorate, our goal of examining methylation defects in the context of spontaneous decidualization required exceptionally pure sources of stromal cells [44]. We anticipate that this will provide a clear framework as we explore methylation in more complex samples, such as endometriotic cells from tissues other than endometrioma, and also the eutopic endometrium in women with endometriosis, where the populations of cells with altered DNA methylation will likely represent only a small fraction of the sample.

A challenge in our work and to this field in general, is the difficulty in demonstrating and quantifying the influence of DNA methylation on gene expression. The lack of methods for experimentally manipulating CpG methylation in a site-specific manner prevents us from unambiguously interrogating the direct effects of these epigenetic marks. This problem is exacerbated by the complexity that arises when multiple methylation differences are observed across a region. Are some regions more influential than others? What mechanisms govern how methylation will be correlated with gene transcription? Most work addressing these questions has relied on correlating DNA methylation with respect to the genomic context in which it occurs. Early observations identifying CGIs near gene promoters led to the prediction that the methylation of these regions silenced downstream genes [45], and this convention is important in cancer where hypermethylation of CGIs is frequently observed against a backdrop of global hypomethylation [46]. Growing evidence suggests this model to be over simplistic, in particular in non-cancerous cells, as CGIs more often escape methylation, while more isolated CpGs within the genome are more variably methylated, and better correlated with gene expression [47], [48]. Moreover, these variable regions do not always demonstrate a negative correlation with gene expression, as we observed for genes such as *NR5A1*, the *HOXA* cluster, and the *ZFPMs*, and further studies are needed to identify possible mechanisms at work. Often intronic enhancers or repressors are epigenetically regulated by methylation. This is the case for *NR5A1* which is differentially expressed in the adrenal, the hypothalamus, and the gonad under the direction of multiple elements [27], [49], [50]. The intronic regions of *NR5A1* are hypermethylated in the adrenal as well as in endometriotic stromal cells, which allows for higher levels of expression [26]. More recently, intragenic methylation has been show to alter tissue-specific transcription factor and methyl CpG binding protein MECP2 in order to alter tissue-specific splicing and gene expression [51], [52].

The differential methylation we uncovered in endometriosis suggests that several different mechanisms are at work. Unlike cancer cells, there was significant hypo- and hypermethylation in endometriotic cells, and these were distributed in a variety of patterns. The majority of differential methylation was observed intragenically and at sites distal to CGIs. While the gene-centric bias in the array may explain the over-representation of intragenic CpGs, the array was similarly biased in favor of island CpGs, suggesting the increased incidence in differential methylation across shore and open sea regions is biologically relevant. Moreover, shore and open sea CpGs were much more likely to be negatively correlated with gene expression, particularly when they occurred near the TSS (such as the TSS200 and 1stExon groups). Recent work from several groups suggests that intragenic methylation, in particular near the first exon, is important for coordinating tissue-specific nucleosome positioning and gene expression [53], [54]. Although the well-spaced coverage of the 450K beadchip makes it possible to detect many of these differences, larger data sets are needed for the extrapolation of unique methylation patterns which correlate with gene expression[55]. We anticipate that our present data will continue to provide valuable insight into gene regulation in endometriosis as future studies decode the spatial and genomic context through which DNA methylation can affected transcription.

Given the limitations we faced in correlating the effects of methylation on gene expression, we developed a novel interaction modeling pathway to better capitalize on the broad number of probe sets provided on the 450K beadchip. The rationale for this was to improve the fidelity with which we could identify the genes whose expression was associated with differential methylation. This model accurately identified genes either known or suspected to be affected by DNA methylation in endometriosis, such as *ESR2*, *NR5A1*, *PGR* and *HOXA10* [3], [4], [56]. While the array we utilized extensively expands these findings by providing more detailed and quantified differences, our model focused on identifying regions with more extensive and significant deviations in methylation (the HOXA cluster for example). Similarly, our model identified genes that are frequently affected by aberrant methylation in other diseases such as cancer, including the tumor suppressors deleted in cancer 1 (*DLC1*) and transcription factor 21 (*TCF21*). The most exciting finding from this model was the large number of novel transcription factors, such as *ESR1* and the *HOXC* cluster. Methylation of *ESR1* frequently silences its expression in cancer, but our discovery of aberrant *ESR1* methylation in endometriosis is novel [57], [58]. Endometriotic cells typically have increased levels of ESR2 due to hypomethylation of its promoter, and the interplay between ESR1 and ESR2 results in the altered response of the diseased cells to estrogen [59].

**Figure 12:**
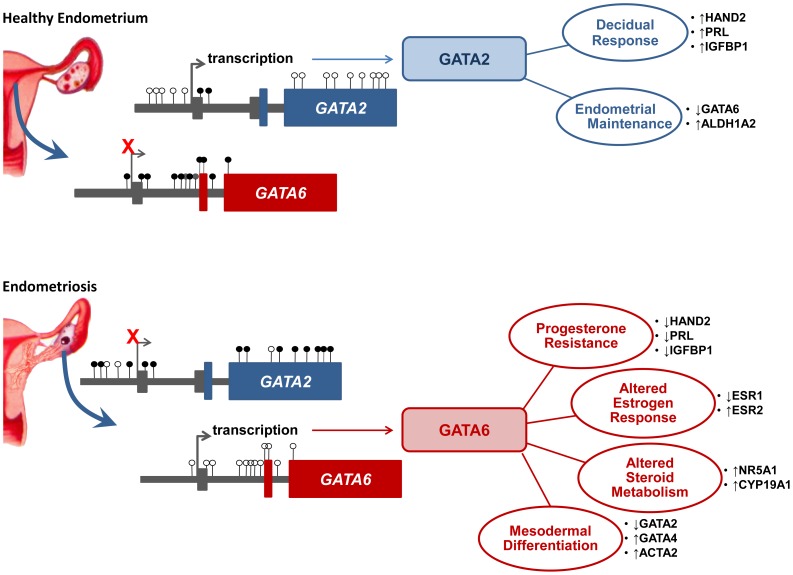
A putative epigenetic switch in endometriosis. In EIUM the GATA2 gene is characterized by a lack of DNA methylation. In contrast, OSIS cells have hypermethyated CpGs throughout a CGI and its proximal shores in the GATA2 promoter, as well as in shore regions throughout the body of the gene. Multiple regions of GATA6 are fully methylated in EIUM, while OSIS cells show a lack of methylation at CpGs across a set of three CGIs and their shores, all located within the body of the gene. We propose this pattern permits GATA2 expression in healthy cells, which coordinates the upregulation of many genes during IVD, and may also maintain the expression of ALDH1A2, a key enzyme in retinoid metabolism. The altered methylation in diseased cells allows GATA6 to be expressed, possibly at the expense of GATA2. GATA6 regulates the expression of multiple gene sets, including those involved in steroid metabolism, the nuclear steroid hormone receptors, and the other GATA family members. Importantly, GATA6 drives EIUM away from the normal endometrial phenotype that is capable of spontaneous decidualization, and into a pattern of gene expression similar to endometriosis.

The most surprising discovery in the list of differentially methylated genes was the GATA family of transcription factors, as the physiological role of this family in the uterus is largely unknown. In particular, the unique roles of GATA2 and GATA6 in apparent opposition to each other, is novel in the uterus (Figure 12). We expected GATA4 may also serve in conjunction with GATA6, since GATA4 has been detected in rabbit endometrium [60] and is often seen to function alongside GATA6; however, our results suggest GATA4 is much less abundant in human EIUM and OSIS. Several studies have also demonstrated a role for *GATA3* in the endometrium throughout the menstrual cycle and in women with endometriosis where it may play a role in modulating cytokine expression [61], [62]; however its expression and methylation were not statistically different based on our arrays. More recently, GATA2 was identified in the mouse endometrium where it appears to coordinate PGR signaling in decidualizing stroma [63], fitting well with our results.

We identified GATA2 to be dominant in EIUM where it may be important for spontaneous decidualization, as either silencing GATA2 or overexpressing GATA6 can disrupt the effects of IVD. Notably, GATA2 knockdown in EIUM did not affect steroid hormone receptor expression or the expression of many of the genes on our panel, but disrupted the early targets of progesterone, such as *HAND2* and *IGFBP*. Thus GATA2 may be important in amplifying signal transduction from maternal progesterone; however additional work is necessary to determine how GATA2 expression is affecting decidualization. Additionally, we saw that exogenous GATA2 in OSIS was able to promote the expression of *ALDH1A2*. Retinoid synthesis is important for endometrial function, whereas reduced retinoid synthesis contributes to increased survival and decreased apoptosis in endometriosis [24], [64]. Additionally GATA2 was able to antagonize *GATA6* and concomitantly *CYP19A1* in OSIS. While we do not know what predisposes GATA6 to be expressed in OSIS, is conceivable that GATA2 could help mitigate improper GATA6 expression. Thus in addition to enhancing progesterone sensitivity, GATA2 may be important in maintaining the unique differentiated state of the endometrial stroma. The role of GATA2 needs to be further studied with respect to decidualization, and in vivo models in mouse and non-human primates would be ideal for characterizing its function during the estrous cycle and menstrual cycle, respectively.

The overexpression of GATA6 drives the gene expression profile in EIUM toward that seen in OSIS, which appear to only express GATA6. Given this dramatic phenotype, it was remarkable that the depletion of GATA6 and exogenous GATA2 expression did not reverse the OSIS phenotype. This is likely a consequence of the more significant methylation defects that have accrued in the diseased cells, which render them unable to respond properly to steroid hormones. For example, even with GATA2, the lack of PGR in OSIS cells would still prohibit effective decidualization. Based on its role in erythrocyte differentiation, GATA2 is thought to promote growth and stem-like properties of progenitor cells, and its transcriptional activity is opposed by GATA1 [65]. In this model, GATA2 binds and drives its own promoter, but the induction of GATA1 is able to supplant and inhibit GATA2 directly on the chromatin, creating a “GATA switch.” It remains to be seen if a similar switch exists in EIUM and OSIS, as we did not examine the occupancy of GATA sites, nor have we demonstrated conclusively that methylation is responsible for maintaining repression of the GATAs in these cells. Likewise, it will be very exciting to determine if GATA isoform expression can affect DNA methylation patterns across the regions we have studied here. However, the idea of an epigenetic switch is provocative, especially for *GATA6*, which demonstrates a strikingly inverted pattern of methylation based on β-values. From this perspective, it is also remarkable that GATA2 is expressed in the healthy endometrium; just like the erythrocyte progenitors, a substantial pool of endometrial stromal cells must be maintained between menstrual cycles, suggesting that GATA2 might also be important for ensuring a population of stromal cells is retained each cycle.

GATA6 is crucial during the earliest stages of embryogenesis, and serves as a multifaceted differentiator in endoderm- and mesoderm-derived tissues [66]–[68]. Additionally, it directly enhances the *ACTA2* promoter [69], which helps explain the strong ACTA2 signals we observed *in vitro*, and likely explains some of the ACTA2 typically seen *in vivo* in endometriotic lesions [70], [71]. Interestingly, ACTA2 shows a complex cyclic pattern of expression in different layers of the baboon and human endometrium, and it will be interesting to examine if GATA6 expression is coordinated in distinct zones of eutopic endometrium [72]–[74]. GATA6 is also a key regulator of many of the steroidogenic enzymes in the gonad, including aromatase [75]. We and others have shown that steroid signaling is altered in endometriotic cells, where loss of DNA methylation allows the expression of aromatase and other enzymes necessary to synthesize estrogen endogenously, locking them in a progesterone-resistant, estrogen-primed state that promotes their survival and growth [76], [77]. Our observation that GATA6 increased *NR5A1* and *CYP19A1* in EIUM suggests that it may be instrumental in allowing endometriotic cells to become steroidogenic. We were very excited to notice that many of the same genes induced by GATA6 are normally repressed by methylation in OSIS. Could the different GATA transcription factors be involved in remodeling the methylation of the genes that drive endometriosis?

During knockdown experiments we noticed that siRNA-mediated loss of the endogenous GATA isoform did not result in increased expression of other isoforms. Similarly, and as mentioned above, the expression of *NR5A1* and other nuclear receptors remained unchanged in OSIS after GATA6 knockdown and GATA2 expression, suggesting that their methylation pattern was fixed and/or was coordinated through other mechanisms. This fits with the mounting evidence suggesting that de novo DNA methylation does not initiate gene silencing, but instead occurs secondarily at genes that have already been transcriptionally silenced [78]. In such a scenario repressors and other factors that may in include GATA family members may serve to silence gene expression, and these loci would then subsequently methylated by recruited DNMTs. A possible example is GATA6 which has CGI in its proximal promoter that is not differentially methylated (Figure 5K), and is uniformly unmethylated based on the data from our array. This region is densely populated by conserved transcription factor binding sites that coordinate its tissue-specific expression, and includes several conserved GATA binding sites [45], [68]. It is plausible that tissue or even disease-specific factors able to bind this region can serve to coordinate the expression and methylation of the downsteam regions.

The more important experiment will be to address the methylation status and expression of *GATA6* and *NR5A1* in the eutopic endometrium of women with endometriosis. This will be challenging, as the methylation defects may be rare occurrences in otherwise healthy stroma; however, our preliminary findings show increased *GATA6* and *NR5A1*, as well as their downstream targets *CYP19A1* and the steroidogenic acute regulatory protein *STAR* ([50], [79]and unpublished observations). It will be very interesting to examine where and how GATA2 and GATA6 compete across the promoters of these genes, and if they can differentially affect DNMT recruitment.

The significant differences in DNA methylation that we detected for genes such as *GATA6* may also be suitable for use as a marker for endometriosis. A conclusive diagnosis of endometriosis can only be made histologically using surgically excised lesions [80]–[82]. Diagnosis is often further complicated as the pain and other symptoms of endometriosis are shared with many other conditions [2]. These problems often cause the definitive diagnosis of endometriosis to lag behind the advent of its symptoms [83]. The ability to use differential DNA methylation as a biomarker is rapidly evolving [84], and would be immensely useful if developed as a sensitive and minimally invasive test for endometriosis. We anticipate that continued progress along this topic will reveal an epigenetic fingerprint for endometriosis that will shed insight both on the origin of the disease, and also open up new approaches for detecting the disease.

## Materials and Methods

### Ethics statement

The acquisition of human tissue for this study was approved by the Northwestern Institutional Review Board for Human Research (1375–005). Written, informed consent from each subject was obtained before surgery.

### Human tissue collection

Normal, eutopic endometrial tissue was obtained from subjects without endometriosis (average age 42.6±5.1 years) undergoing hysterectomy for benign conditions (cervical dysplasia or uterine leiomyoma). Ectopic endometrium from the cyst walls of ovarian endometriomas was obtained immediately after surgery (average age 41.3±4.6 years). All patients were premenopausal and underwent surgery during the proliferative phase of their menstrual cycle, having received no preoperative hormonal therapy. Endometriosis was confirmed for each sample by histological examination.

### Isolation and culture of primary stromal cells

Enzymes for tissue processing were obtained from Sigma (St. Louis, MO). Cell culture media, trypsin, and supplements were from Gibco (Life Technologies, Carlsbad, CA). Cell plastics were from TPP (St. Louis, MO). Homogenous populations of primary stromal cells were isolated from eutopic endometrial tissue and from endometriotic tissue as previously described [85], [86]. Briefly, stroma and glandular fragments were dissected from adjacent tissue, minced, and digested with collagenase and DNase at 37°C for 30 min. Samples were then treated with collagenase, DNase, pronase, and hyaluronidase at 37°C for an additional 30 min. Epithelial cells were eliminated by progressive filtration through sterile 70- and 20-µm sieves, and either human endometrial stromal cells (EIUM) or endometriotic stromal cells (OSIS) were dispensed into 100-mm dishes for adherent growth and maintained in DMEM/F12 supplemented with 10% fetal bovine serum, 100 IU/mL penicillin, 100 µg/mL streptomycin, and 2.5 µg/mL amphotericin B. Cells were maintained in grown in a humidified atmosphere with 5% CO_2_ at 37°C, and medium was replenished every 48 h.

### *In vitro* decidualization

*In vitro* decidualization (IVD) regimens followed previous protocols [40]. Briefly, both normal and diseased stromal cells were grown to ∼75% confluency, then switched to phenol red-free DMEM/F12 media supplemented with 2% charcoal-dextran-stripped FBS and antibiotics as above. Controls were maintained in this reduced medium, while IVD treatment consisted of 1 µM medroxyprogesterone acetate (MPA; Sigma), 35 nM 17β-estradiol (E2; Sigma), and 0.05 mM 8-bromoadenosine 3′,5′-cyclic monophosphate (BIOLOG Life Science Institute, Bremen Germany). Cells underwent 6-day IVD treatments following gene silencing or overexpression. All other IVD treatments were conducted for 10 days.

### Transfection and transduction

For knockdown experiments, 400,000 EIUM/60-mm dish were transiently transfected with Silencer Select siRNAs from Ambion (Life Technologies) using Lipofectamine RNAiMAX (Life Technologies). Individual siRNAs targeting *GATA2* or *GATA6* (Table S3) were tested individually for specificity, and then optimized for transfection as a pair. On the day of transfection, EIUM were trypsinized and resuspended in antibiotic-free DMEM/F12 with 10% FBS, while siRNAs were prepared per the manufacturer's protocol for reverse transcription to yield a final concentration of 50 nM siRNA in 4.4 mL of medium/dish (0.22 nMol of total siRNA in complex with 15 µL of reagent). Cells were replaced with complete media 12 hours post-transfection, and IVD treatments were initiated 36 hours post-transfection, and continued for 6 days.

For adenoviral transduction, 400,000 EIUM/60-mm dish (or 30,000 per well in 24-well dishes) were infected in complete medium, at an MOI of 20 with adenoviral particles (Vector Biolabs, Philadelphia, PA) carrying either enhanced GFP (AdGFP), human *GATA2* (AdGATA2), or human *GATA6* (AdGATA6), each under direction of the CMV promoter. IVD treatments were initiated 24 hours post-infection (at which point GFP expression was visually confirmed), and continued for 6 days. (For combined transfection/transduction siRNAs were transfected first, transduction followed 12 hours as cells were transferred to complete media, and IVD treatments were started 24 hours after transduction).

### Cell imaging and immunofluorescence

For staining and visualization, stromal cells were cultured on sterile 12-mm, #1.5 glass coverslips (Thermo Fisher Scientific, Hampton, NH) in 24-well dishes as described above. Cells were fixed in 1x PBS with 4% formaldehyde (Electron Microscopy Sciences, Hatfield, PA), and then either stained with hematoxylin and eosin (VWR), or for individual proteins using indirect immunofluorescence as described previously, but with minor modifications [87]. Briefly, after permeabilization and washing, fixed cells were washed twice with 1x TBS-T (20 mM Tris-HCl, 500 mM NaCl, pH 7.4, 0.05% Tween-20), and then blocked in TBS with 1% nonfat milk and 1% normal donkey serum (Jackson Immunoresearch Inc., West Grove, PA). Primary antibodies (Table S3) were prepared in blocking solution (1∶50 dilution) and incubated for 1 h. Secondary antibodies conjugated to Cy-3 (Jackson Immunoresearch) were prepared in blocking solution (1∶200 dilution), and incubated for 1 h. DNA was stained using DAPI, and the coverslips were then washed twice with TBS. Mounted coverslips were examined under brightfield or epifluorescence settings with a Zeiss Axiovert 200 using a 40x LDPlan-NEOFLUAR or a 63x Plan-APOCHROMATIC objective, and images were acquired using an Axiocam HRc.

### Nucleic acid isolation and beadchip arrays

For microarray analyses, total genomic DNA and total RNA from each plate of cells were isolated using AllPrep DNA/RNA columns (Qiagen, Valencia, CA). DNA quality was assessed by visualization following agarose gel electrophoresis. To selectively convert unmethylated cytosine to uracil, one microgram of genomic DNA was subjected to bisulfite treatment using EZ DNA Methylation kits (Zymo, Orange, CA), and either frozen or then followed by isothermal amplification according to the manufacturer's protocol (Illumina, San Diego, CA). The converted genomic DNA was then directly hybridized to Infinium HumanMethylation450 beadchips, and scanned using the Illumina iScan system. Total RNA quality was assessed using an Agilent Bioanalyzer 2100, and 1 µg of high quality RNA (RIN>9) from each subject was hybridized to HT-12v4 beadchips, and also scanned on the iScan system. Image data were processed in Genome Studio. The analysis of raw data was done with an in-house analysis pipeline described in the *Data analysis* subsection.

### Reverse transcription and qPCR

For analysis of samples following transfection or transduction, total RNA was isolated using RNeasy columns (Qiagen). Total cDNA was prepared with Q-script cDNA SuperMix (Quanta Biosciences, Gaithersburg, MD) [88]. Real-time PCR was performed as previously described, using Power SYBR green or Taqman Universal master mix, on an ABI 7900 (Applied Biosystems, Foster City, CA, USA) [89]. Relative gene expression was assessed using TATA-binding protein (*TBP*) as a reference gene. For endogenous expression before and after treatment, fold change was calibrated to the average ΔC_T_ of untreated EIUM. For analysis of samples following transfection or transduction, average fold change was calculated after calibrating to each subject's untreated negative control. Primer information is provided in Table S3.

### Methylation specific PCR

To validate the methylation status of GATA2 and GATA6 we employed methylation specific PCR using the following primers directed against differentially methylated regions of exon 4 of *GATA2*, and exon 2 of *GATA6*. Briefly, primers were designed to recognize either the methylated or unmethylated form of the sequence after the bisulphite converted sequences of the “-“ strand. Primer information is provided in Table S3. Each MSP reaction utilized 25 ng of bisulfite converted DNA from either EIUM or OSIS as template. For controls, we used female genomic DNA that was either fully methylated (M) and bisulfite converted or fully demethylated (U) and bisulfite converted. Each reaction was carried out in a 20 µL volume containing 500 nM of each primer. Reactions proceeded using a hot start of 95C for 5 minutes, followed by 38 cycle, 3-step reaction using a melt at 95C for 1 min, annealing at 58C for 1 min, and an extension at 72C for 1 min. Amplification products were resolved on 3% agarose gel, and visualized with ethidium bromide under UV light.

### Preparation of protein and immunoblotting

Whole cell lysates were prepared by washing cells with PBS, followed by lifting and homogenizing the cells in 120 µL RIPA buffer (50 mM Tris pH 7.6, 150 mM NaCl, 0.1% SDS, 0.5% sodium deoxycholate, 1% NP-40) supplemented with protease inhibitor cocktail (Sigma). Lysates were cleared by centrifugation at 14,000× g for 5 min. Equal amounts of protein (20 µg) were resolved on NuPAGE Novex 4–12% bis-Tris Gels (Life Technologies). Transfer and membrane blocking were performed as previously described [90]. Incubation with primary antibodies (Table S3) was performed at 4°C in 2.5% nonfat milk overnight. The membranes were then washed and incubated with the appropriate HRP-conjugated secondary antibodies for 1 h. Detection was performed using Luminata Crescendo HRP substrate (Millipore).

### Data analysis

We developed a pipeline in R/Bioconductor [91] to integrate the analysis of methylation and mRNA microarrays (under submission). The mRNA data were preprocessed to eliminate probes associated to genes in the X and Y chromosomes. The raw probe intensities were then converted to expression values by firstly applying a variance-stabilizing transformation within each chip and, secondly, by performing a robust spline normalization between chips. The signal preprocessing was conducted in R using the lumi library [92]. Differentially expressed probes were identified between phenotypes (6 samples each) using empirical Bayes correction of linear models provided by the limma library [93], [94]. The *p*-values of probes were adjusted for multiple-hypotheses testing using the Benjamini-Hochberg algorithm. Probes with an adjusted *p*-value less than 0.05 were considered differentially expressed. Differentially expressed probes were then mapped to unique transcripts (RefSeq IDs). If multiple probes mapped to the same RefSeq ID, then only the probe with the smallest adjusted *p*-value was kept. Probes that did not map to a known RefSeq ID were discarded.

For methylation data, probes mapping to the X and Y chromosomes or to non-CpGs dinucleotides were excluded. Two-color data from the methylation array were normalized using quantile normalization. Probes were marked as “present” if their detectable probe ratio was greater than 0.01. A probe was discarded if it was present in 4 or fewer samples of the 6 samples for each phenotype. The probe intensities were then converted to β-values. As it was the case for mRNA data, the methylation two-color data were preprocessed with the lumi library. A CpG probe was considered to be differentially methylated if the (absolute) difference between the average β-value for one phenotype (6 samples) was greater than 0.15 with respect to the average β-value of the other phenotype (6 samples), i.e., Δβ>0.15. The average β-values in a phenotype were obtained from the probes marked as present in that phenotype. Differentially methylated CpG probes were linked to differentially expressed transcripts using the definitions in the chip manifests provided by the manufacturer.

A Spearman rank correlation coefficient (ρ) was obtained between each differentially methylated CpG and its matched differentially expressed transcript, based on the β- values of the CpG and the expression values of the transcript across samples. The sign of ρ was used to classify CpGs as being positively or negatively correlated to the transcripts. For ρ>0 we had two cases: hypermethylation and over-expression, or hypomethylation and under-expression; Likewise, for ρ<0 we had hypermethylation and under-expression, or hypomethylation and over-expression.

A test of proportion was conducted on each type of CpG location, stratified either by gene or CGI. The total proportion of negatively over positively correlated CpGs was compared against the proportions of negatively over positively correlated CpGs at each type of location. Two-tailed tests were conducted under the null hypothesis that the proportions at each location were equal to the total proportion.

ANOVA interaction analysis was conducted for each differentially expressed transcript on the β-values of all the CpGs that were linked to the transcript. The interaction that was tested was between CpG status (hypo- vs. hypermethylated) and CpG location. As it was the case before, two different types of locations were used to determine the interaction with CpG status: location with respect to the transcript or with respect to the closest CGI. The location context for each CpG was taken from the manufacturer's manifest. Importantly, Illumina stratifies the regions flanking CGIs as either “shore” or “shelf” (depending on proximity) as well as being either north or south of the island depending on their orientation with respect to the starting coordinate for the chromosome. For simplicity, and because we only wished to stratify these regions relative to genes and CGIs, we merged the shelf and shores into a single category defined as a region 4 kb upstream or downstream of their most proximal CGI. Genes were considered to have a statistically significant interaction if the adjusted *p*-value for this test was less than 0.05.

MetaCore (version 6.13 build 43450, Thomson Reuters) was used to perform enrichment analysis workflows on data matched to each target RefSeq ID. The “Enrichment by Protein Function” tool was used to perform over-representation analysis (ORA) based on protein class for the genes identified by ANOVA interaction analysis. To identify biological processes uniquely altered during IVD that were likely to be affected by differentially methylated genes, the “Compare Experiments Workflow” tool was used on the ANOVA interaction list and the list of differentially expressed genes in OSIS versus EIUM after IVD. Enrichment analysis was performed on an intersection of both groups' gene ontology processes, and then sorted and ranked where overlap was most similar.

### Statistical analysis

For qPCR data, differences across experimental/diseased groups treated with and without IVD were assessed by two-way ANOVA. When no significant interaction was present, a main effect was considered significant for a *p*-value of less than (0.05). When an interaction was detected, multiple comparisons were made using Tukey's test. Differences in GATA isoform expression between EIUM and OSIS were made using t-tests.

## Supporting Information

Figure S1 The expression of ZFPM1 and ZFPM2 in EIUM and OSIS is shown using (A) qPCR (asterisks indicate *p*<0.05, t-test, n = 5), (B) immunoblot for 4 EIUM and 4 OSIS samples.
(PDF)

Figure S2 Representative methylation specific PCR for GATA2 and GATA6 in EIUM and OSIS. MSP primers were designed to cover differentially methylated regions in exon 4 of GATA2 and in exon 2 of GATA6. These regions contained CpGs that were identified as differentially methylated using the HumanMethylation450 beadchip. The methylation status being interrogated by each set of primers used is indicated across the. Templates are labeled across the bottom: 100%U is fully unmethylated, bisulfite converted control template; 100%M is fully methylated, bisulfite converted control template; EIUM and OSIS indicate samples of bisulfite converted genomic DNA from stromal cells taken from 4 different subjects.
(PDF)

Table S1 Differentially expressed genes identified from HT-12v4 beadchip. Tab 1. OSIS- vs EIUM-. Tab 2. OSIS+ vs EIUM+. Tab 3. EIUM+ vs EIUM−. Tab 4. OSIS+ vs OSIS−. Tab 5. Genes identified by ANOVA interaction.
(XLSX)

Table S2 Differentially methylated CpGs identified from Infinium HumanMethylation450 beadchip. Tab 1. OSIS− vs EIUM−. Tab 2. EIUM+ vs EIUM−. Tab 3. OSIS+ vs OSIS−. Tab 4. OSIS− vs EIUM− CpGs matched to differentially expressed genes in same group. Tab 5. EIUM+ vs EIUM− CpGs matched to differentially expressed genes in same group. Tab 6. CpGs matched to genes identified by ANOVA interaction.
(XLSX)

Table S3 Lists of reagents used. Tab 1. DNA oligo primers and probes used for qPCR and MSP. Tab 2. siRNA oligos used for transient transfection. Tab 3. Antibodies used for immunoblotting and immunofluorescence.
(XLSX)
