# Supplementary figures and images for: Genome-Wide DNA Methylation Analysis Predicts an Epigenetic Switch for GATA Factor Expression in Endometriosis
Source: PLoS Genet. 2014 Mar 6;10(3):e1004158. doi: 10.1371/journal.pgen.1004158 (PMC3945170; doi:10.1371/journal.pgen.1004158)

**A)**

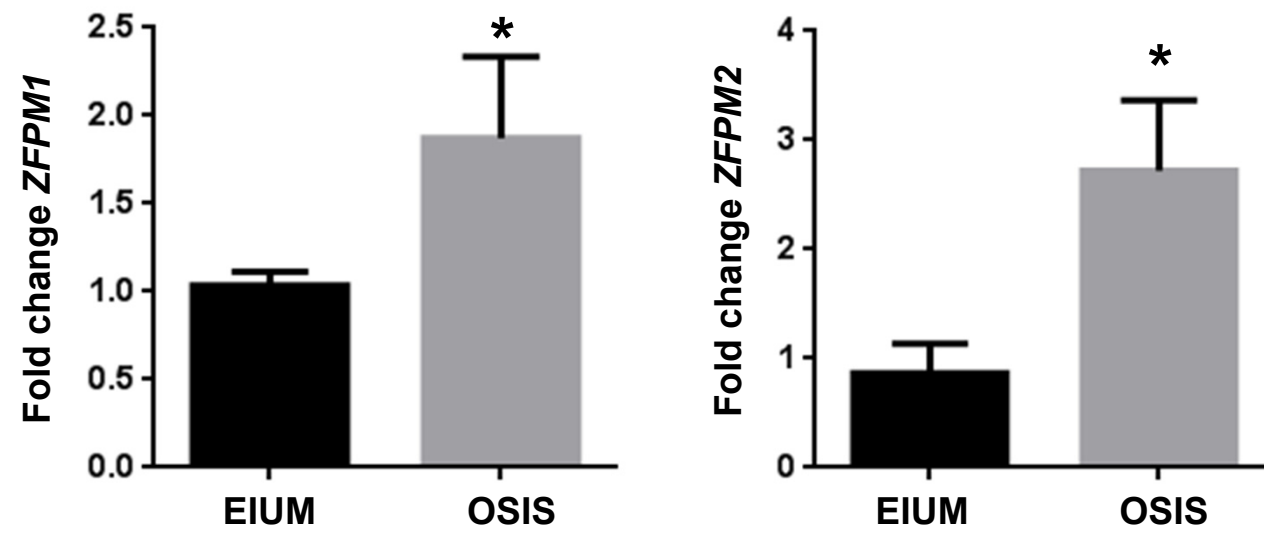

**B)**

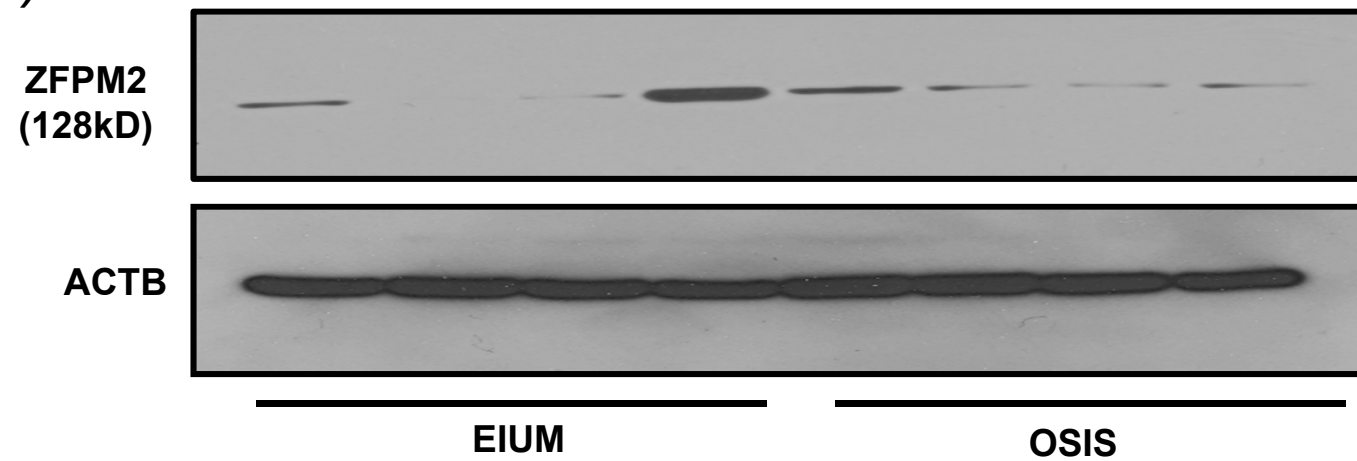

Supplement: Figure S1 — The expression of ZFPM1 and ZFPM2 in EIUM and OSIS is shown using (A) qPCR (asterisks indicate p<0.05, t-test, n = 5), (B) immunoblot for 4 EIUM and 4 OSIS samples. (PDF) [file pgen.1004158.s001.pdf]

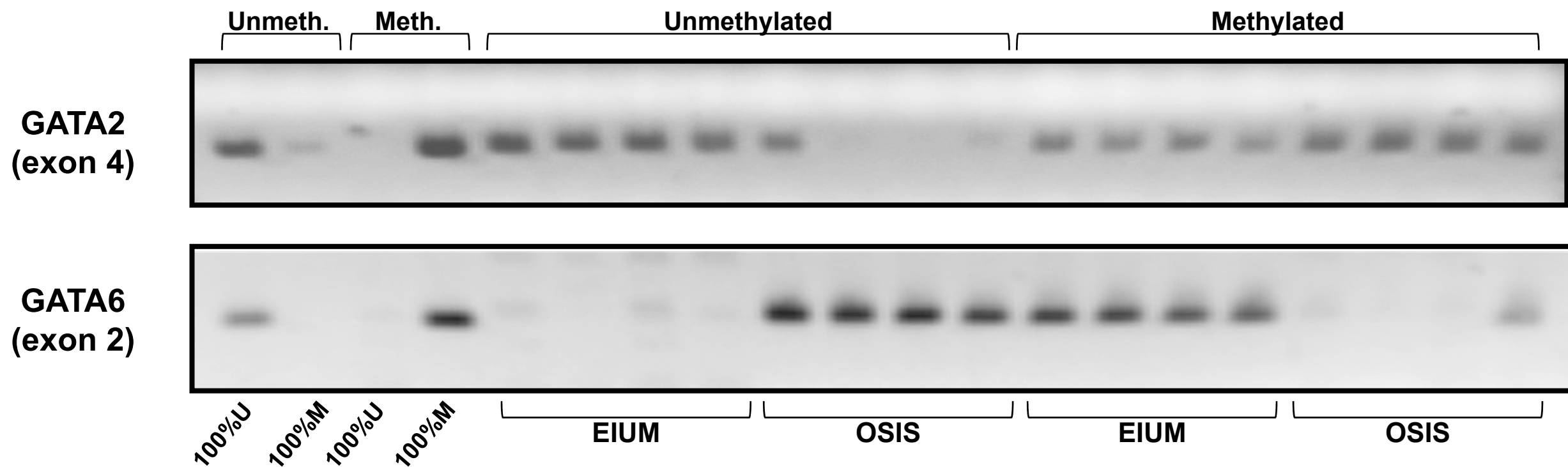

Supplement: Figure S2 — Representative methylation specific PCR for GATA2 and GATA6 in EIUM and OSIS. MSP primers were designed to cover differentially methylated regions in exon 4 of GATA2 and in exon 2 of GATA6. These regions contained CpGs that were identified as differentially methylated using the HumanMethylation450 beadchip. The methylation status being interrogated by each set of primers used is indicated across the. Templates are labeled across the bottom: 100%U is fully unmethylated, bisulfite converted control template; 100%M is fully methylated, bisulfite converted control template; EIUM and OSIS indicate samples of bisulfite converted genomic DNA from stromal cells taken from 4 different subjects. (PDF) [file pgen.1004158.s002.pdf]
